# Psychological status of infertile men during the Coronavirus Disease 2019 Pandemic in China: a cross-sectional investigation

**DOI:** 10.1186/s12610-022-00177-5

**Published:** 2023-02-16

**Authors:** Zhe Zhang, Yu-Zhuo Yang, Hai-Tao Zhang, Yu Xi, Cun-Tong Wang, De-Feng Liu, Jia-Ming Mao, Hao-Cheng Lin, Wen-Hao Tang, Lian-Ming Zhao, Xian-Sheng Zhang, Yu-Tian Dai, Hui Jiang

**Affiliations:** 1 Department of Urology, Peking University Third Hospital, Beijing, China; 2 Department of Reproductive Medicine Center, Peking University Third Hospital, Beijing, China; 3 School of Social Development, Central University of Finance and Economics, Beijing, China; 4 Department of Urology, The First Affiliated Hospital of Anhui Medical University, Hefei, China; 5 Department of Andrology, Nanjing Drum Tower Hospital, Nanjing, China

**Keywords:** COVID-19, Male infertility, Psychological health, Sexual function, COVID-19, Infertilité masculine, Santé psychologique, Fonction sexuelle

## Abstract

**Background:**

The coronavirus disease 2019 (COVID-19) outbreak has had a widespread and profound impact on people’s mental health. The factors associated with mental symptoms among men diagnosed with infertility, a disease closely related to psychological conditions, remain unclear. The aim of this study is to investigate the risk factors associated with mental symptoms among infertile Chinese men during the pandemic.

**Results:**

A total of 4,098 eligible participants were recruited in this cross-sectional, nationwide study, including 2,034 (49.6%) with primary infertility and 2,064 (50.4%) with secondary infertility. The prevalence of mental health conditions was 36.3%, 39.6%, and 6.7% for anxiety, depression, and post-pandemic stress, respectively. Sexual dysfunction is associated with a higher risk with adjusted odds ratios (ORs) of 1.40 for anxiety, 1.38 for depression, and 2.32 for stress. Men receiving infertility drug therapy displayed a higher risk for anxiety (adjusted OR, 1.31) and depression (adjusted OR, 1.28) symptoms, while those receiving intrauterine insemination had a lower risk of anxiety (adjusted OR, 0.56) and depression (adjusted OR, 0.55) symptoms.

**Conclusion:**

The COVID-19 pandemic has had a significant psychological impact on infertile men. Several psychologically vulnerable populations were identified, including individuals with sexual dysfunction, respondents receiving infertility drug therapy, and those experiencing control measures for COVID-19. The findings provide a comprehensive profile of the mental health status of infertile Chinese men during the COVID-19 outbreak and provide potential psychological intervention strategies.

**Supplementary Information:**

The online version contains supplementary material available at 10.1186/s12610-022-00177-5.

## Résumé

**Contexte:**

L’épidémie de maladie à coronavirus 2019 (COVID-19) a eu un impact étendu et profond sur la santé mentale des gens. Les facteurs associés aux symptômes mentaux chez les hommes diagnostiqués comme infertiles, une maladie étroitement liée aux conditions psychologiques, restent flous. L’objectif de cette étude est d’étudier les facteurs de risque associés aux symptômes mentaux chez les hommes chinois infertiles pendant la pandémie.

**Résultats:**

Au total, 4 098 participants admissibles ont été recrutés dans cette étude transversale à l’échelle nationale, dont 2 034 (49,6%) présentaient une infertilité primaire et 2 064 (50,4%) une infertilité secondaire. La prévalence des problèmes de santé mentale était respectivement de 36,3 %, 39,6 % et 6,7 % pour l’anxiété, la dépression, et le stress postpandémique. La dysfonction sexuelle est associée à un risque plus élevé avec des odds ratios ajustés (OR) de 1,40 pour l’anxiété, 1,38 pour la dépression et 2,32 pour le stress. Les hommes recevant un traitement médicamenteux contre l’infertilité présentaient un risque plus élevé de symptômes d’anxiété (OR ajusté, 1,31) et de dépression (OR ajusté, 1,28), alors que ceux dont le traitement consistait à faire des inséminations intra-utérines présentaient un risque plus faible de symptômes d’anxiété (OR ajusté, 0,56) et de dépression (OR ajusté, 0,55).

**Conclusions:**

La pandémie de COVID-19 a eu un impact psychologique important sur les hommes infertiles. Plusieurs populations psychologiquement vulnérables ont été identifiées, notamment les personnes souffrant de dysfonction sexuelle, les hommes recevant un traitement médicamenteux contre l’infertilité, et ceux subissant des mesures de contrôle de la COVID-19. Les résultats fournissent un profil complet de l’état de santé mentale des hommes Chinois infertiles pendant l’épidémie de COVID-19 et fournissent des stratégies potentielles d’intervention psychologique.

## Background

The coronavirus disease 2019 (COVID-19) outbreak occurred in December 2019 and arousedhas attracted global attention [1]. According to WHO, which declared COVID-19 as a pandemic, the disease has infected more than 318 million people and caused more than six million deaths worldwide across more than 200 countries, areas, or territories as of October 2022 [2]. The daily life of most was significantly changed owing to the pandemic control measures. A series of measures were enforced in China, including restrictions on transport, entertainment, and social distancing measures [3–5]. Studies have shown that during a global pandemic, individuals may experience psychological problems [6] such as anxiety, depression, posttraumatic stress disorder, and negative societal behaviors such as societal rejection, discrimination, and stigmatization [7]. COVID-19 has had a serious impact on psychological and sexual health, as well as interpersonal relationships among the general population [6], especially in patients with stroke [8], hypertension [9], and Parkinson’s disease [10].

Infertility refers to the incapacity to conceive after one year of regular, unprotected sexual intercourse, and affects approximately one in five couples worldwide [11–13]. Male factors contribute to almost half of all cases, and approximately 7% of men worldwide are afflicted with this condition [14]. Indeed, it has been widely documented that being infertile significantly impacts the psychological well-being of both partners, who experience problems such as low self-esteem, sexual distress, depression, guilt, anxiety, frustration, and relational issues between themselves. Additionally, several studies have reported that these negative emotions can also be detrimental to sexual health and relationship quality, even conception, creating a vicious cycle [15]. For men, potential inverse dose-response relationships were revealed between anxiety [16] and depression [17] with sperm quality (concentration, motility, and total sperm count). Mental health symptoms are closely related to male sexual dysfunction [18–20], which is an important cause of male infertility. Infertility treatment is closely related to a patient’s psychological condition, and dealing with infertility has a significant impact on their well-being and life satisfaction. These may originate from two underlying causes: fertility is related to patients’ interpersonal (social, relational, and marital) experiences, and it is connected to the importance of parenthood in a couple’s life [21–23].

The COVID-19 outbreak has had a widespread and profound impact on people’s psychological status, leading to the emergence of new mental symptoms or deterioration of existing mental illnesses [24–26]. Simultaneously, ongoing infertility treatment and medical support may be interrupted and delayed [27–29]. Infertile couples may feel isolated and neglected during this outbreak period, and may withdraw from their family and friends. A few studies have explored the impact of the pandemic on the mental health of female patients with infertility, and participants reported more negative emotions, including stress, worry, and frustration [27, 30, 31]. Infertile men can also experience a heavy psychological burden, especially under pressure from Chinese tradition and social role identification. Thus, to provide evidence-based guidance and help for infertile men during the COVID-19 pandemic, we conducted a large-sample cross-sectional study to investigate the mental health condition of Chinese infertile men.

## Methods

### Study design

This cross-sectional study was conducted from September 4–21, 2020. A stratified cluster random sampling method was employed to recruit participants (Supplementary Fig. 1), and the sample was obtained from the sampling frame of seven categories developed by stratifying all geographical regions. Sampling followed a tiered process that included three levels: provincial-level administrative regions, cities, and hospitals. Two representative provinces were randomly selected in each of the central and eastern regions (considering population density): one representative province was randomly selected in each of the other geographical regions, and the provincial capital city, together with two random secondary cities, were chosen in these provinces. Four tertiary hospitals and four secondary hospitals (two in the provincial capital city and one in each secondary city) were selected. Next, excessive sampling was conducted in Beijing, Shanghai, and Guangdong provinces, considering the economic level, urban size, and population density. Four tertiary and four secondary hospitals were selected from the three provincial-level administrative regions. Outpatient physicians involved in patient recruitment at all hospitals were trained to improve data collection consistency. Couples who had not been able to conceive a child even though they had frequent, unprotected sexual intercourse for a year or longer were recruited in the urology/andrology department and reproductive center. The husband’s personal, sexual, and medical/medication history were documented, after which he underwent a physical examination, semen analysis, and hormonal evaluation. Men with sperm disorders, hypogonadism, ejaculation disorders, or other factors affecting infertility were included in this study. Men with mental retardation or other diseases leading to an inability to understand the questionnaire items were excluded from this study. Written informed consent was obtained before the respondents completed the questionnaire.

### Questionnaires

The survey comprised four parts. The first part gathered the demographic information of the participants, including age, education level, occupation, chronic disease and psychiatric disorder history, and sleeping status. The second part inquired about infertility status. The third part investigated pandemic-related conditions and attitudes toward the COVID-19 pandemic; we inquired about the COVID-19 infection history of participants and/or people around them, quarantine experiences, being frontline workers, and attitudes about COVID-19 affecting male reproduction. Information on these three parts is listed in Supplementary Table 1.

The fourth part of the questionnaire comprised five standardized scales, including the Chinese versions of the International Index of Erectile Function-5 items (IIEF-5) [32], Premature Ejaculation Diagnostic Tool (PEDT) [33], Generalized Anxiety Disorder-7 (GAD-7) [34], Patient Health Questionnaire-9 (PHQ-9) [35], and Impact of Event Scale-Revised (IES-R) [36], which measure symptoms of erectile dysfunction (ED), premature ejaculation (PE), anxiety, depression, and post-pandemic stress. The total scores of these scales were interpreted as follows: IIEF-5, normal (22–25), mild (17–21), and severe to moderate (5–16) erectile dysfunction (ED); PEDT, normal (0–8), suspicious (9–10), and confirmed (11–20) premature ejaculation (PE); GAD-7, normal (0–4), mild (5–9), moderate (10–14), and severe (15–21) anxiety; PHQ-9, normal (0–4), mild (5–9), moderate (10–14), and severe (15–21) depression; IES-R, normal (0–33); confirmed (34–88). In this study, cut-off scores of 5 for the GAD-7, 5 for the PHQ-9, and 34 for the IES-R were adopted to detect symptoms of anxiety, depression, and post-pandemic stress.

### Statistical analysis

Continuous variables are presented as means and standard deviations. Categorical variables are represented as absolute and percentage frequencies. All analyses were conducted using SPSS 20.0 (SPSS Inc., Chicago, IL, USA). All continuous variables were evaluated for normal distribution using the Shapiro-Wilk test; non-continuous variables with non-normal distribution of variance were evaluated using the Mann-Whitney U-test. Comparison of proportions was evaluated using the chi-square test. The Bonferroni method was used to adjust *P*-values when comparing the proportions. To explore factors potentially associated with anxiety, depression, and stress, unadjusted logistic regression and multivariable logistic regression analyses were performed. Odds ratios (ORs) and 95% confidence intervals (CIs) are presented. All statistical tests were two-tailed, and the significance level was set at *P* < 0.05.

## Results

### Demographic information

A total of 4,450 men were recruited from 96 urology/andrology and reproductive centers. After screening the incomplete questionnaires, data from 4,098 eligible participants were included in the final analysis, including 2,034 (49.6%) with primary infertility and 2,064 (50.4%) with secondary infertility. Of the total sample, 1,988 (48.5%) had been diagnosed with infertility for less than three years, 1,289 (31.5%) had been diagnosed for three years or more, and 821 (20.0%) had an uncertain infertility history. The mean age was 32.65 ± 6.32 years old; 2,629 (64.2%) respondents possessed a college degree or higher; and 2,687 (65.6%) lived in urban areas. A total of 60.4% (2,476 cases) lived in a nuclear family and 80.3% (3,289 cases) held a steady job. More than half of the participants (2,150 men, 52.5%) had an average of more than eight hours of sleep per night during the pandemic, and 834 participants (20.4%) reported at least one sleeping disorder. Infertile men with ED and PE—the most common sexual dysfunction disorders—accounted for 57.1% and 15% of men, respectively. This survey included data from 61 individuals (1.5%) with confirmed or suspected cases of COVID-19, 30 (0.7%) individuals had been in close contact with COVID-19 patients, and 504 (12.3%) were frontline workers. A total of 793 (19.3%) participants underwent quarantine, and 2,705 (66.0%) reported that their daily work was affected by COVID-19. Additional demographic and pandemic-related characteristics are presented in Supplementary Tables 2 and 3.

### Prevalence of anxiety, depression, and post-pandemic stress symptoms

**Fig. 1:**
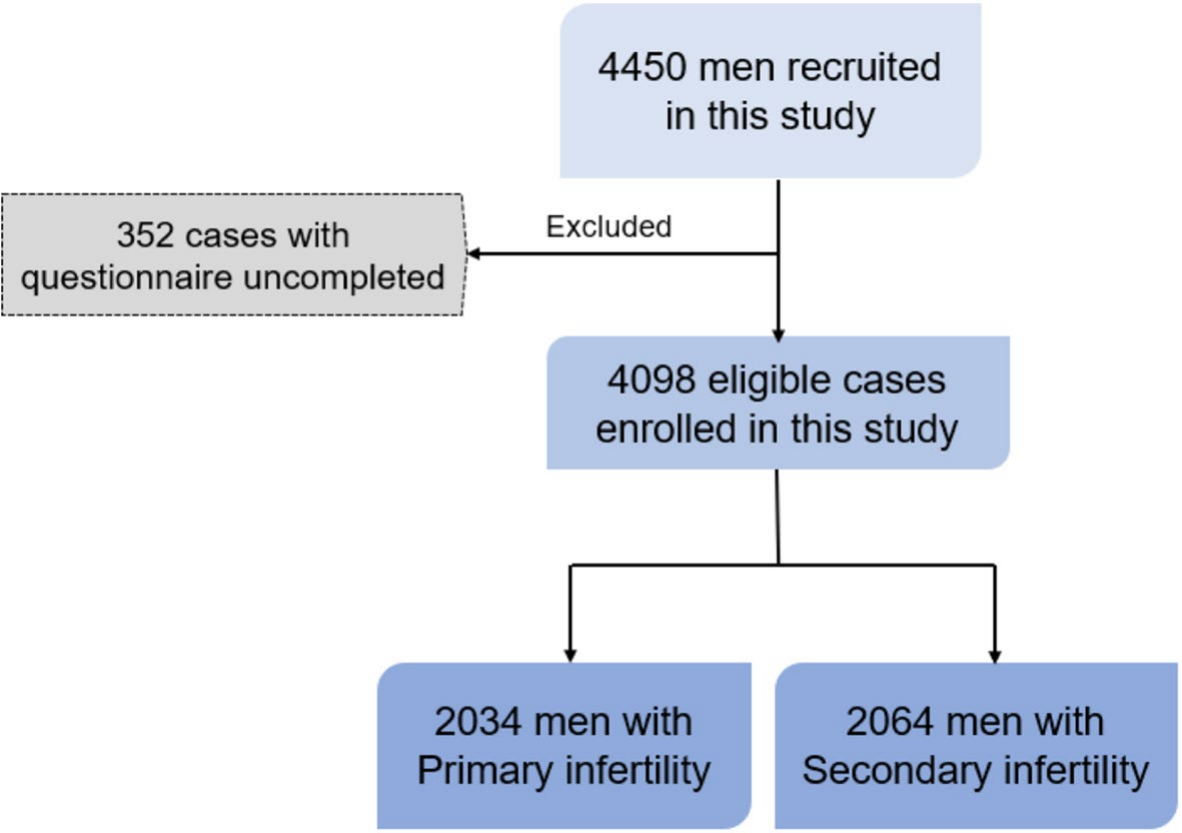
Flowchart of the study

**Fig. 2:**
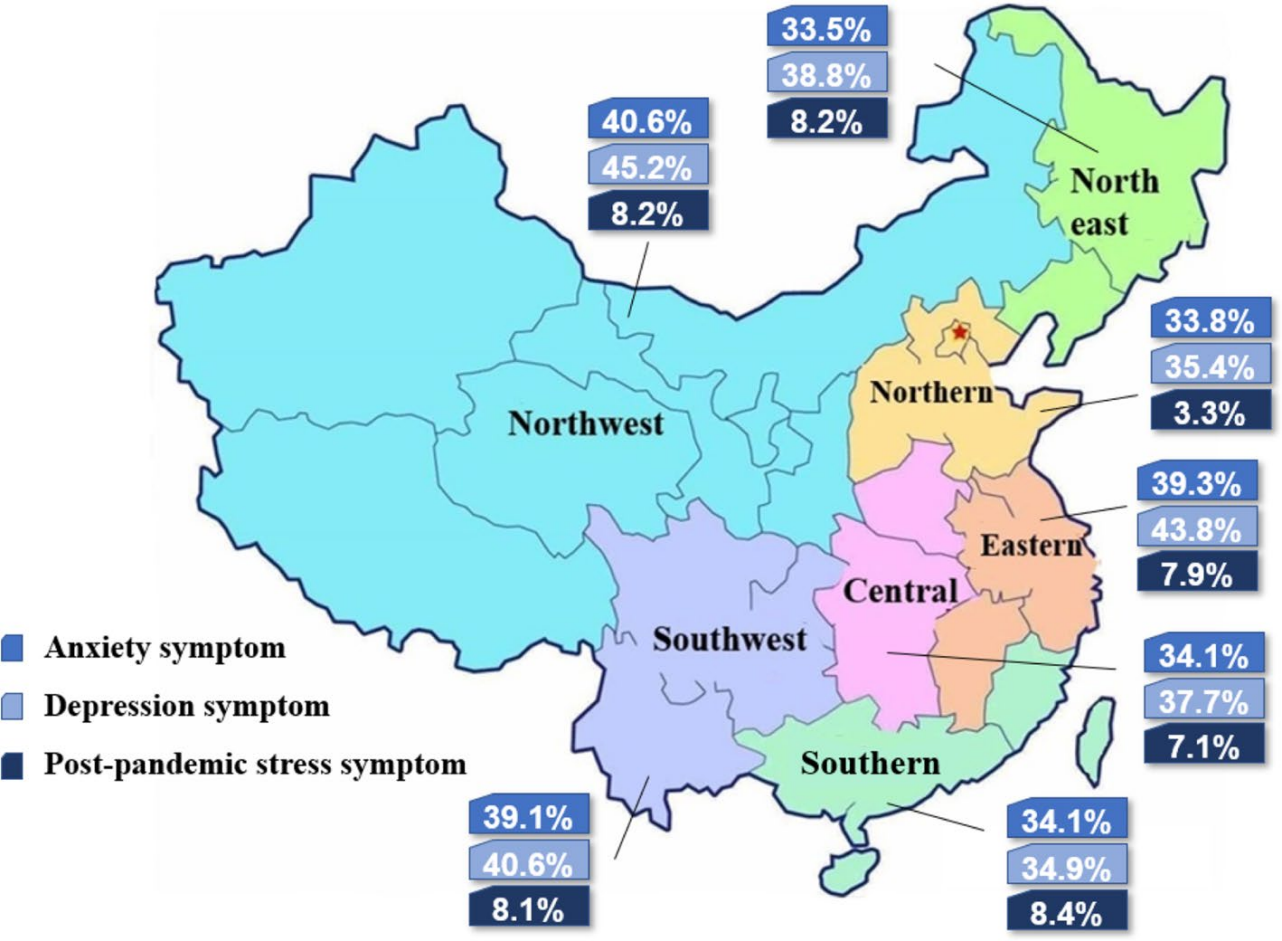
Prevalence of anxiety, depression, and post-pandemic stress symptoms in seven geographical regions of China

In this study, 1,884 men (46.0%) had at least one psychological symptom, 1,011 (24.7%) had two different symptoms, and 244 (6.0%) had three different symptoms. The prevalence of symptoms for psychological conditions among infertile men was 36.3% (95% CI, 34.8–37.7%) for anxiety (1,486 participants total, including 1,139 participants [27.8%] with mild anxiety and 347 participants [8.5%] with moderate-to-severe anxiety), 39.6% (95% CI, 38.1–41.1%) for depression (1,623 participants total, including 1,149 participants [28.0%] with mild depression and 474 participants [11.6%] with moderate-to-severe depression), and 6.7% (95% CI, 5.9–7.5%) for post-pandemic stress (274 participants). The prevalence of anxiety (39.3%), depression (43.8%), and post-pandemic stress symptoms (7.9%) in eastern China was significantly higher than that in other areas of China, while depression and post-pandemic stress were significantly lower in northern China (35.4% and 3.3%, respectively). The prevalence of anxiety, depression, and post-pandemic stress symptoms in the different geographical regions is shown in Figs. 1 and 2.

Among 1,486 men with anxiety symptoms, 1,021 (68.7%) had ED symptoms and 495 (33.3%) had PE symptoms. In those with depressive symptoms (1,623 men), 1,125 (69.3%) had ED symptoms and 549 (33.8%) had PE symptoms. The percentage of participants with post-pandemic stress symptoms increased to 78.8% (ED, 216 men) and 48.9% (PE, 134 men). Of the 994 men with moderate to severe ED symptoms, 146 (14.7%) had moderate to severe anxiety symptoms, 209 (21.0%) had moderate to severe depressive symptoms, and 132 (13.3%) had stress symptoms, as measured by the validated questionnaires. Among the 613 men with PE symptoms, 116 (18.9%) had moderate to severe anxiety symptoms, 157 (25.6%) had moderate to severe depressive symptoms, and 92 (15.0%) had stress symptoms. The prevalence of psychological symptoms was high among men with insomnia (anxiety, 66.5%; depression, 72.7%; stress, 19.8%), sleep duration less than eight hours (anxiety, 41.7%; depression, 45.1%; stress, 8.0%), men on medication (anxiety, 42.9%; depression, 46.0%; stress, 7.6%), frontline workers (anxiety, 42.1%; depression, 48.6%), participants who experienced both centralized quarantine (anxiety, 42.9%; depression, 52.4%) and home quarantine (anxiety, 42.6%; depression, 48.6%), participants with working delayed (anxiety, 38.8%; depression, 43.6%; stress, 7.4%), participants out of work (anxiety, 36.5%; depression, 39.9%; stress, 12.2%), participants experiencing salary cuts or unemployment during COVID-19 (anxiety, 43.4%; depression, 47.1%; stress, 8.5%), and participants reporting increase in workload (anxiety, 43.7%; depression, 47.3%; stress, 9.0%). Meanwhile, the prevalence of psychological symptoms was also high among men with the following conditions: fever, fatigue, or headache (anxiety, 53.1%; depression, 70.3%; stress, 14.1%), worrying about being infected (anxiety, 44.2%; depression, 48.4%; stress, 9.9%), being concerned about the impact of COVID-19 on sexual function (anxiety, 49.5%; depression, 53.3%; stress, 15.7%), and choosing cryopreservation of sperm in response to COVID-19 (anxiety, 44.4%; depression, 44.6%; stress, 13.2%). The prevalence of psychological problems among the different populations is presented in Table 1.

**Table 1:** Prevalence of Symptoms of Anxiety, Depression and Post-pandemic stress in Population with Personal History and Different Pandemic Experience

Factors	Anxiety ^a^				Depression ^b^				Post-pandemic stress ^c^	
	Mild n (% ^d^)	Moderate to severe n (%^d^)	Total n (%^d^) [95% CI]	*P* ^e^	Mild n (%^d^)	Moderate to severe n (%^d^)	Total n (%^d^) [95% CI]	*P* ^e^	Total n (%^d^) [95% CI]	*P* ^e^
**Level of education**										
Junior high school or below	126(2.0%)	57(9.9%)	183(31.9%) [28.2-35.8%]	0.001	125(21.8%)	74(12.9%)	199(34.7%) [30.9-38.7%]	0.002	43(7.5%) [5.6-9.9%]	0.536
Senior high school or technical secondary school	222(24.8%)	71(7.9%)	293(32.7%) [29.7-35.8%]		227(25.3%)	104(11.6%)	331(36.9%) [33.8-40.1%]		54(6.0%) [4.6-7.7%]	
College degree or higher	791(30.1%)	219(8.3%)	1010(38.4%) [36.6-40.3%]		797(30.3%)	296(11.3%)	1093(41.6%) [39.7-43.5%]		177(6.7%) [5.8-7.7%]	
**Job status**										
Erratic	224(27.7%)	94(11.6%)	318(39.3%) [36.0-42.7%]	0.044	220(27.2%)	130(16.1%)	350(43.3%) [39.9-46.7%]	0.018	62(7.7%) [6.0-9.6%]	0.214
Steady	915(27.8%)	253(7.7%)	1168(35.5%) [33.9-37.2%]		929(28.2%)	344(10.5%)	1273(38.7%) [37.1-40.4%]		212(6.4%) [5.6-7.3%]	
**History of psychiatric disorders**										
No	1107(27.6%)	322(8.0%)	1429(35.6%) [34.1-37.1%]	< 0.001	1124(28.0%)	440(11.0%)	1564(38.9%) [37.4-40.5%]	< 0.001	258(6.4%) [5.7-7.2%]	< 0.001
Yes	32(39.0%)	25(30.5%)	57(69.5%) [59.0-78.7%]		25(30.5%)	34(41.5%)	59(72.0%) [61.6-80.8%]		16(19.5%) [12.1-29.1%]	
**Sleep disorders**										
No	824(25.2%)	228(7.0%)	1052(32.2%) [30.6-33.8%]	< 0.001	847(25.9%)	293(9.0%)	1140(34.9%) [33.3-36.6%]	< 0.001	166(5.1%) [4.4-5.9%]	< 0.001
Insomnia	121(43.5%)	64(23.0%)	185(66.5%) [60.9-71.9%]		106(38.1%)	96(34.5%)	202(72.7%) [67.2-77.6%]		55(19.8%) [15.4-24.8%]	
Snoring	159(33.1%)	41(8.5%)	200(41.7%) [37.3-46.1%]		172(35.8%)	58(12.1%)	230(47.9%) [43.5-52.4%]		40(8.3%) [6.1-11.1%]	
Both	20(40.8%)	13(26.5%)	33(67.3%) [53.5-79.2%]		12(24.5%)	23(46.9%)	35(71.4%) [57.8-82.6%]		13(26.5%) [15.8-40.0%]	
Others	15(55.6%)	1(3.7%)	16(59.3%) [40.6-76.1%]		12(44.4%)	4(14.8%)	16(59.3%) [40.6-76.1%]		0(0.0%) -	
**Sleeping duration/per night**										
< 8 h	515(31.1%)	174 (10.5%)	689(41.7%) [39.3-44.0%]	< 0.001	514(31.1%)	232(14.0%)	746(45.1%) [42.7-47.5%]	< 0.001	133(8.0%) [6.8-9.4%]	< 0.001
≥ 8 h	561(26.1%)	151(7.0%)	712(33.1%) [31.2-35.1%]		573(26.7%)	210(9.8%)	783(36.4%) [34.4-38.5%]		128(6.0%) [5.0-7.0%]	
Variable	63(21.4%)	22(7.5%)	85(28.9%) [24.0-34.3%]		62(21.1%)	32(10.9%)	94(32.0%) [26.8-37.5%]		13(4.4%) [2.5-7.2%]	
**Ongoing treatment**										
None	614(25.5%)	203(8.4%)	817(34.0%) [32.1-35.9%]	< 0.001	632(26.3%)	264(11.0%)	896(37.3%) [35.3-39.2%]	< 0.001	149(6.2%) [5.3-7.2%]	< 0.001
Medication	420(34.0%)	111,9.0%	531(42.9%) [40.2-45.7%]		407(32.9%)	162(13.1%)	569(46.0%) [43.2-48.8%]		94(7.6%) [6.2-9.2%]	
Surgical treatment	25(28.7%)	9(10.3%)	34(39.1%) [29.3-49.6%]		22(25.3%)	17(19.5%)	39(44.8%) [34.7-55.3%]		12(13.8%) [7.8-22.2%]	
Others	80(21.7%)	24(6.5%)	104(28.2%) [23.8-32.9%]		88(23.8%)	31(8.4%)	119(32.2%) [27.6-37.1%]		19(7.5%) [4.7-11.2%]	
**ED symptom**										
None	376(21.4%)	89(5.1%)	465(26.4%) [24.4-28.5%]	< 0.001	385(21.9%)	113(6.4%)	498(28.3%) [26.2-30.4%]	< 0.001	58(3.3%) [2.5-4.2%]	< 0.001
Mild	416(31.0%)	112(8.3%)	528(39.3%) [36.7-41.9%]		437(32.5%)	152(11.3%)	589(43.9%) [41.2-46.5%]		84(6.3%) [5.1-7.6%]	
Moderate to severe	347(34.9%)	146(14.7%)	493(49.6%) [46.5-52.7%]		327(32.9%)	209(21.0%)	536(53.9%) [50.8-57.0%]		132(13.3%) [11.3-15.5%]	
**PE symptom**										
None	804(26.3%)	187(6.1%)	991(32.5%) [30.8-34.1%]	< 0.001	818(26.8%)	256(8.4%)	1074(35.2%) [33.5-36.9%]	< 0.001	140(4.6%) [3.9-5.4%]	< 0.001
Suspected	138(31.9%)	44(10.2%)	182(42.1%) [37.5-46.8%]		132(30.6%)	61(14.1%)	193(44.7%) [40.0-49.4%]		42(9.7%) [7.2-12.8%]	
Yes	197(32.1%)	116(18.9%)	313(51.1%) [47.1-55.0%]		199(32.5%)	157(25.6%)	356(58.1%) [54.1-61.9%]		92(15.0%) [12.3-18.0%]	
**Did you get fever, fatigue or headache during pandemic?**										
No	1114(27.6%)	338(8.4%)	1452(36.0%) [34.5-37.5%]	0.005	1116(27.7%)	462(11.5%)	1578(39.1%) [37.6-40.6%]	< 0.001	265(6.6%) [5.8-7.4%]	0.017
Yes	25(39.1%)	9(14.1%)	34(53.1%) [41.0-65.0%]		33(51.6%)	12(18.8%)	45(70.3%) [58.4-80.4%]		9(14.1%) [7.2-24.1%]	
**Are you a frontline worker?**										
No	979(27.2%)	295(8.2%)	1274(35.4%) [33.9-37.0%]	0.004	962(26.8%)	416(11.6%)	1378(38.3%) [36.8-39.9%]	< 0.001	238(6.6%) [5.8-7.5%]	0.661
Yes	160(31.7%)	52(10.3%)	212(42.1%) [37.8-46.4%]		187(37.1%)	58(11.5%)	245(48.6%) [44.3-53.0%]		36(7.1%) [5.1-9.6%]	
**Have you ever experienced quarantine?**										
No	879(26.6%)	269(8.1%)	1148(34.7%) [33.1-36.4%]	< 0.001	873(26.4%)	365(11.0%)	1238(37.5%) [35.8-39.1%]	< 0.001	216(6.5%) [5.7-7.4%]	0.051
Centralized	19(30.2%)	8(12.7%)	27(42.9%) [31.2-55.2%]		22(34.9%)	11(17.5%)	33(52.4%) [40.2-64.4%]		9(14.3%) [7.3-24.4%]	
Home	241(33.0%)	70(9.6%)	311(42.6%) [39.1-46.2%]		254(34.8%)	98(13.4%)	352(48.2%) [44.6-51.8%]		49(6.7%) [5.1-8.7%]	
**How was your work affected by the pandemic?**										
None	290(20.8%)	70(5.0%)	360(25.8%) [23.6-28.2%]	< 0.001	294(21.1%)	94(6.7%)	388(27.9%) [25.5-30.3%]	< 0.001	47(3.4%) [2.5-4.4%]	< 0.001
Delayed	250(29.7%)	77(9.1%)	327(38.8%) [35.6-42.2%]		252(29.9%)	115(13.7%)	367(43.6%) [40.3-47.0%]		62(7.4%) [5.7-9.3%]	
Kept jobless	29(19.6%)	25(16.9%)	54(36.5%) [29.1-44.4%]		26(17.6%)	33(22.3%)	59(39.9%) [32.2-47.9%]		18(12.2%) [7.6-18.1%]	
Salary cut or job loss	471(32.8%)	153(10.6%)	624(43.4%) [40.8-46.0%]		476(33.1%)	202(14.0%)	678(47.1%) [44.6-49.7%]		122(8.5%) [7.1-10.0%]	
Workload increased	99(35.7%)	22(7.9%)	121(43.7%) [37.9-49.6%]		101(36.5%)	30(10.8%)	131(47.3%) [41.5-53.2%]		25(9.0%) [6.1-12.8%]	
**Are you worried about you and your relatives infected by coronavirus?**										
No	643(24.6%)	189(7.2%)	832(31.8%) [30.0-33.6%]	< 0.001	655(25.0%)	251(9.6%)	906(34.6%) [32.8-36.4%]	< 0.001	127(4.9%) [4.1-5.7%]	< 0.001
Yes	496(33.5%)	158(10.7%)	654(44.2%) [41.7-46.7%]		494(33.4%)	223(15.1%)	717(48.4%) [45.9-51.0%]		147(9.9%) [8.5-11.5%]	
**Are you concerned that infection with coronavirus may affect your sexual function?**										
No	844(26.0%)	221(6.8%)	1065(32.8%) [31.2-34.4%]	< 0.001	868(26.7%)	301(9.3%)	1169(36.0%) [34.4-37.7%]	< 0.001	140(4.3%) [3.7-5.1%]	< 0.001
Yes	295(34.7%)	126(14.8%)	421(49.5%) [46.1-52.8%]		281(33.0%)	173(20.3%)	454(53.3%) [50.0-56.7%]		134(15.7%) [13.4-18.3%]	
**Have you considered choosing cryopreservation of sperm in response to the COVID-19 pandemic?**										
No	980(27.2%)	288(8.0%)	1268(35.2%) [33.6-36.7%]	< 0.001	1009(28.0%)	395(11.0%)	1404(38.9%) [37.3-40.5%]	0.016	209(5.8%) [5.1-6.6%]	< 0.001
Yes	159(32.4%)	59(12.0%)	218(44.4%) [40.0-48.8%]		140(28.5%)	79(16.1%)	219(44.6%) [40.2-49.0%]		65(13.2%) [10.5-16.4%]	

Abbreviation: ED, erectile dysfunction; PE, premature ejaculation; COVID-19, coronavirus disease 2019

^a^Scores of 5 to 9 on the Generalized Anxiety Disorder–7 were defined as mild anxiety, and scores of 10 or higher were defined as moderate-to-severe anxiety

^b^Scores of 5 to 9 on the Patient Health Questionnaire–9 were defined as mild depression, and scores of 10 or

higher were defined as moderate-to-severe depression

^c^Post-pandemic stress was defined as Impact of Event Scale-Revised score of 33 or higher

^d^The proportion of different symptom-severity subgroups to each risk-factor subgroup

^e^χ2 tests were used to compare the prevalence of mild-to-severe mental health symptoms in different populations

### Factors associated with symptoms of anxiety, depression, and post-pandemic stress

In the multivariable analysis, participants with a college or higher educational background and history of psychiatric disorders displayed a remarkably higher risk of anxiety and depression symptoms. Sleeping disorders, ED, and PE were found to be associated with symptoms of anxiety, depression, and stress, and participants with sleep duration longer than eight hours had a lower risk of anxiety (adjusted OR, 0.84; 95% CI, 0.73–0.97) and depression (adjusted OR, 0.86; 95% CI, 0.74–1.00) symptoms. Simultaneously, being concerned about the impact of COVID-19 on sexual function is associated with a higher risk with adjusted ORs of 1.40 (95% CI, 1.18–1.67) for anxiety, 1.38 (95% CI, 1.16–1.64) for depression, and 2.29 (95% CI, 1.70–3.08) for post-pandemic stress symptoms. Men receiving infertility drug therapy displayed a higher risk of anxiety (adjusted OR, 1.30; 95% CI, 1.12–1.52) and depression (adjusted OR, 1.27; 95% CI, 1.09–1.48) symptoms. Men considering cryopreservation of sperm in response to the pandemic had an elevated risk of stress symptoms (adjusted OR, 1.50; 95% CI, 1.06–2.10). Participants whose work was affected by COVID-19 faced higher risks than those whose work was not affected. Among these impacts, salary cut/job loss was associated with at least twice the risk of anxiety, depression, and stress symptoms (adjusted ORs, 2.02 [95% CI, 1.70–2.41] for anxiety, 2.16 [95% CI, 1.81–2.57] for depression, and 2.01 [95% CI, 1.39–2.89] for stress symptoms). Men out of work were highly susceptible to symptoms of stress (adjusted OR, 3.64; 95% CI, 1.98–6.70). Men worried about COVID-19 infection demonstrated a higher risk with adjusted ORs of 1.41 (95% CI, 1.22–1.64) for anxiety, 1.33 (95% CI, 1.13–1.55) for depression, and 1.53 (95% CI, 1.15–2.03) for stress symptoms. Infertile men with a higher desire to receive psychological counseling during the pandemic demonstrated a higher risk of anxiety (adjusted OR, 1.10; 95% CI, 1.08–1.13), depression (adjusted OR, 1.09; 95% CI, 1.06–1.11), and stress (adjusted OR, 1.15; 95% CI, 1.11–1.20). Nonetheless, men with more knowledge about COVID-19 displayed a lower risk of anxiety (adjusted OR, 0.94; 95% CI, 0.91–0.98) and depression (adjusted OR, 0.95; 95% CI, 0.91–0.98). Other risk factors for depressive symptoms included fever, fatigue, or headache during the pandemic (adjusted OR, 2.26; 95% CI, 1.22–4.21), being frontline workers (adjusted OR, 1.36; 95% CI, 1.09–1.70), and home quarantine experiences (adjusted OR, 1.29; 95% CI, 1.08–1.55). Detailed results of the multivariable analysis are presented in Table 2.

**Table 2:** Multivariable Regression Analysis of Risk Factors Associated with Symptoms of Anxiety, Depression, and Post-pandemic Stress

Factors	Anxiety ^a^		Depression ^b^		Post-pandemic stress ^c^	
	AOR ^d^ [95% CI]	*P*	AOR ^d^ [95% CI]	*P*	AOR ^d^ [95% CI]	*P*
**Level of education**						
Junior high school or below	1 [Reference]		1 [Reference]		NA	
Senior high school or technical secondary school	1.10 [0.86–1.40]	0.443	1.18 [0.93–1.50]	0.175		
College degree or higher	1.79 [1.45–2.22]	< 0.001	1.80 [1.45–2.22]	< 0.001		
**History of psychiatric disorders**						
No	1 [Reference]		1 [Reference]		NA	
Yes	2.64 [1.58–4.43]	< 0.001	2.50 [1.47–4.28]	0.001		
**Sleep disorders**						
No	1 [Reference]		1 [Reference]		1 [Reference]	
Insomnia	3.10 [2.34–4.11]	< 0.001	3.62 [2.69–4.87]	< 0.001	3.64 [2.52–5.25]	< 0.001
Snoring	1.39 [1.13–1.72]	0.002	1.60 [1.30–1.98]	< 0.001	1.70 [1.15–2.50]	0.007
Both	2.97 [1.56–5.66]	0.001	3.07 [1.57-6.00]	0.001	4.66 [2.26–9.61]	< 0.001
Others	2.42 [1.09–5.37]	0.030	1.98 [0.88–4.47]	0.098	0.00	0.998
**Sleeping duration/per night**						
< 8 h	1 [Reference]		1 [Reference]		NA	
≥ 8 h	0.84 [0.73–0.97]	0.020	0.86 [0.74-1.00]	0.046		
Variable	0.63 [0.47–0.84]	0.002	0.64 [0.48–0.86]	0.003		
**Ongoing treatment**						
None	1 [Reference]		1 [Reference]		1 [Reference]	
Medication	1.30 [1.12–1.52]	0.001	1.27 [1.09–1.48]	0.003	0.97 [0.72–1.30]	0.817
Surgical treatment	1.18 [0.73–1.90]	0.497	1.33 [0.83–2.14]	0.243	2.53 [1.27–5.04]	0.008
Others	0.82 [0.63–1.06]	0.137	0.86 [0.67–1.11]	0.240	0.89 [0.53–1.50]	0.664
**ED symptom**						
None	1 [Reference]		1 [Reference]		1 [Reference]	
Mild	1.58 [1.34–1.86]	< 0.001	1.77 [1.51–2.08]	< 0.001	1.44 [1.00-2.06]	0.049
Moderate to severe	2.15 [1.79–2.57]	< 0.001	2.40 [2.00-2.88]	< 0.001	2.64 [1.86–3.74]	< 0.001
**PE symptom**						
None	1 [Reference]		1 [Reference]		1 [Reference]	
Suspected	1.21 [0.97–1.51]	0.094	1.16 [0.93–1.45]	0.184	1.67 [1.14–2.47]	0.009
Yes	1.56 [1.29–1.90]	< 0.001	1.88 [1.54–2.29]	< 0.001	2.11 [1.54–2.88]	< 0.001
**Did you get fever, fatigue or headache during pandemic?**						
No	NA		1 [Reference]		NA	
Yes			2.26 [1.22–4.21]	0.010		
**Are you a frontline worker?**						
No	NA		1 [Reference]		NA	
Yes			1.36 [1.09–1.70]	0.007		
**Have you ever experienced quarantine?**						
No	NA		1 [Reference]		NA	
Centralized			1.37 [0.78–2.40]	0.279		
Home			1.29 [1.08–1.55]	0.006		
**How was your work affected by the pandemic?**						
None	1 [Reference]		1 [Reference]		1 [Reference]	
Delayed	1.60 [1.31–1.95]	< 0.001	1.79 [1.47–2.19]	< 0.001	1.56 [1.03–2.37]	0.037
Kept jobless	1.56 [1.06–2.29]	0.023	1.73 [1.18–2.53]	0.005	3.64 [1.98–6.70]	< 0.001
Salary cut or job loss	2.02 [1.70–2.41]	< 0.001	2.16 [1.81–2.57]	< 0.001	2.01 [1.39–2.89]	< 0.001
Workload increased	1.76 [1.32–2.34]	< 0.001	1.73 [1.29–2.31]	< 0.001	2.11 [1.23–3.64]	0.007
**Are you worried about you and your relatives infected by coronavirus?**						
No	1 [Reference]		1 [Reference]		1 [Reference]	
Yes	1.41 [1.22–1.64]	< 0.001	1.33 [1.13–1.55]	< 0.001	1.53 [1.15–2.03]	0.003
**Are you concerned that infection with coronavirus may affect your sexual function?**						
No	1 [Reference]		1 [Reference]		1 [Reference]	
Yes	1.40 [1.18–1.67]	< 0.001	1.38 [1.16–1.64]	< 0.001	2.29 [1.70–3.08]	< 0.001
**Have you considered choosing cryopreservation of sperm in response to the COVID-19 pandemic?**						
No	NA		NA		1 [Reference]	
Yes					1.50 [1.06–2.10]	0.021
**How much do you know about COVID-19?**	0.94 [0.91–0.98]	0.001	0.95 [0.91–0.98]	0.004	NA	
**How much do you desire to receive psychological counseling?**	1.10 [1.08–1.13]	< 0.001	1.09 [1.06–1.11]	< 0.001	1.15 [1.11–1.20]	< 0.001

*Abbreviation*: *AOR* Adjusted odds ratio, *N* Not available (variables that were not analyzed because they were not statistically significant in the unadjusted regression model). *ED* Erectile dysfunction, *PE* Premature ejaculation, *COVID-19* Coronavirus disease 2019

^a^Anxiety was defined as Generalized Anxiety Disorder–7 score of 5 or higher

^b^Depression was defined as Patient Health Questionnaire–9 score of 5 or higher

^c^Post-pandemic stress was defined as Impact of Event Scale-Revised score of 33 or higher

^d^All of the variables that were statistically significant in the unadjusted regression analysis and those that might convey important information, including education level, job status, history of psychiatric disorders, sleep disorders, sleeping duration, ongoing treatment, sexual function (ED, PE) and COVID-19 related information were entered into the multivariable model

## Discussion

Severe emotional distress can occur during public health events [37–40]. To the best of our knowledge, numerous studies have examined women with infertility (Table 3). Ceasing infertility treatment was the primary problem affecting patients [27, 41, 42], while changes in working environment and style [43], quarantine [44], and financial concerns [45] increase the psychological burden of patients. Negative emotions were found to reduce the quality of couples’ relationships [28] and lower expectations of future pregnancy [46] during the pandemic. Men with infertility are especially vulnerable to psychological problems [47], and the psychological status of infertile male patients is often overlooked. This is the first nationwide study of an infertile male population that systematically investigated the prevalence of and factors associated with mental health symptoms using standardized rating scales during this COVID-19 outbreak period. This study revealed that the pandemic has had a psychological impact on infertile men, and approximately half of them had at least one psychological symptom. More than one-third of infertile men developed anxiety or depression symptoms, and a certain percentage had post-pandemic stress symptoms. Several psychologically vulnerable populations were identified, including individuals with sexual dysfunction, respondents receiving infertility drug therapy, those with work being affected, those who experienced home quarantine, and frontline workers. The findings provide a comprehensive profile of the mental health status of infertile Chinese men during the COVID-19 outbreak and provide potential psychological intervention strategies.

**Table 3:** Studies about Psychological Status of Infertile population during the Coronavirus Disease 2019 Pandemic

Author(s)	Journal Volume	Study population (infertility)	Sample size	Countries/ regions	Results/conclusions related to psychological status during pandemic
Gordon et al.	PLoS One 2020;15(9)	Females	92	North America	Fertility treatment suspensions have had a considerable negative impact on women’s mental health and quality of life.
Ben-Kimhy et al.	Hum Reprod 2020 12 01;35(12)	Females	168	Israel	Infertility females expressed sadness (64%), helplessness (61%) and distress (50%).
Seifer et al.	Reprod Biol Endocrinol 2021 Feb 23;19(1)	Females and males	734	United States	Mental health, physical health, personal safety, strain on their relationship with their partner and concern regarding their financial situation are the five major concerns during the pandemic.
Lawson et al.	J Assist Reprod Genet 2021 Feb;38(2)	Females and males	787	United States	Distress was induced mostly by the delay of infertility treatments.
Haham et al.	Reprod Biomed Online 2021 04;42(4)	Females	181	Canada	Anxiety related to COVID-19 and disagreement with treatment suspension were found to be significantly associated with psychological distress
Cao et al.	Front Psychiatry 2021;12	Females	1943	China	An increase in negative emotions and worse family relationships among quarantine population.
Rosielle et al.	Reprod Biomed Online 2021 10;43(4)	Females	330	Netherlands	76.6% of the infertility patients reported increased levels of stress during the pandemic.
Dillard et al.	Int J Environ Res Public Health 2022 Feb 23;19(5)	Females	304	United States	An overall negative impact was associated with more negative emotions, lower expectations of future pregnancy, and greater stress and depressive symptoms during the pandemic.
Dong et al.	J Assist Reprod Genet 2022 Feb;39(2)	Female and males	940	China	Anxiety symptoms and stress level are related to the quality of a couple’s relationship.
Galhardo et al.	Psychol Health Med 2022 02;27(2)	Females	89	Portugal	Women who continued to work at their workplace presented significantly higher depressive and anxiety symptoms
Tippett	J Health Psychol 2022 06;27(7)	Females	124	United Kingdom	Increased stress levels due to treatment cancellation has had a detrimental impact on the emotional health and wellbeing of patients.

*Abbreviation*: *COVID-19* Coronavirus disease 2019

The prevalence of anxiety was consistent and depression was higher compared to studies of Chinese infertile men before the COVID-19 outbreak by Gao et al. [48] in 2013 and Ma et al. [20] in 2017. Meanwhile, we found that the prevalence of psychological symptoms was also higher compared to data from the general population during the COVID-19 pandemic [26, 49], which was reported to be approximately one-fifth to one-third. This indicates that the pandemic’s psychological impact is greater on infertile men than on the general population.

As our data showed, several groups were likely to develop psychological symptoms. This study revealed that erection/ejaculation dysfunction leads to a higher risk of mental health symptoms such as anxiety, depression, and stress. PE and ED are two major male sexual dysfunctions with prevalence varying from 20 to 50% in different districts, and the prevalence is even higher among infertile populations [50–54]. The prevalence of ED was reported to increase due to severe semen quality impairment [55]. In daily clinical practice, approximately 50–80% of men with sexual dysfunction show concomitant symptoms of depression or anxiety [18]. Poor mental health can also lead to ED or PE [56–58]. Ejaculatory latency, sexual desire, and orgasmic function are reduced in infertile men and are mainly associated with mood disturbances [55]. The frequency of sexual intercourse was significantly related to changes in erectile function and ejaculatory control ability, and participants with a low frequency of sexual intercourse had less partner time and intimate behavior with sexual partners. Owing to the restrictions on social activity and transport during the pandemic, partners who did not live together had a lower chance of sexual intercourse [59–62]. As two entities that cause and affect each other, sexual function and psychological status must be assessed in infertile populations. A deep analysis of the sexual and psychological status of infertile men, as well as evaluating their association, might be helpful in exploring the effects of infertility on sexual function and possible treatment plans [48], and in improving not only reproductive but also general and sexual health [55].

Drug therapy is usually the first choice in idiopathic male infertility and in men with an abnormality in the hypothalamic-pituitary-testicular axis, leading to endocrine-related infertility [11]. Although drug treatment is a non-invasive and affordable choice, patients still have to pin their hopes on the efficacy of drugs and wait to conceive naturally. Considering the spermatogenesis cycle, the duration of drug treatment is usually 6–12 months, at least [63]. During this pandemic, fertility treatment suspensions have had a considerable negative impact on patients’ mental health and quality of life [41]. Men on medication are likely to experience deterioration of their psychological condition due to a lack of further medical attention.

The secondary social impacts of COVID-19 were identified as boosters for psychological symptoms in this study, which is consistent with a UK-based finding that individuals were more concerned about the secondary social impacts of COVID-19 than the direct threats of COVID-19 infection [64]. Infertile men whose work was affected reported a higher risk for symptoms of anxiety, depression, and post-pandemic stress. As reported in Australia, financial pressure due to salary cut/job loss, which was also commonly identified in our sample, was associated with poorer mental health [65]. Our study also identified home quarantine experience as a risk factor for depressive symptoms, in line with study results from the general population [49, 66]. Owing to the strict policy on social contact, procedures in various industries have slowed down and inevitably leading to delays/backlogs. The lack of office connections might reduce creativity and productivity and is not conducive to the discharge of mental pressure. In China, men are usually the family breadwinners. The negative impact of COVID-19 on work will worsen the situation for men struggling with fertility problems, giving rise to psychological symptoms.

We found that the threat of COVID-19 infection could also contribute to psychological symptoms. A high probability of depressive symptoms was found among frontline workers. People infected with the coronavirus may be asymptomatic during the incubation period, and its clinical manifestations can be easily confused with those of normal influenza [67–69]. During this public health emergency, frontline workers may fear getting sick and spreading the infection to their families, other patients, and coworkers [70]. As our data suggested, men worrying about themselves or their relatives being infected with COVID-19 showed a higher risk of anxiety, depression, and stress, and men with fever, fatigue, or headaches during the pandemic were more vulnerable to depression symptoms. It has been suggested that the coronavirus infection could impair the male reproductive system if spermatogenic tubules, testicular stromal cells, and spermatogenic cells in the testis are invaded by the virus [71–74]. Testicular damage was found to be more severe in men of procreant age than in older men [75–77]. This implies that the potential after-effects of even mild infections are magnified in infertile men and put them under substantial psychological burden. This was further confirmed by our finding that men choosing cryopreservation of sperm in response to the COVID-19 pandemic had a higher risk of stress symptoms.

Sleep conditions may also have influenced mental health during the pandemic. Self-reported insomnia and snoring are the risk factors for anxiety, depression, and stress. The unsettling news and images shown on television and the Internet, together with the uncertainty of the novel virus, might have contributed to the emergence of sleep disorders. Our results demonstrated that snoring can affect both sleep duration and the quality of sleep [78]. Sleep disorders are closely related to anxiety, depression, and stress [79, 80]. Infertile men who claimed to know about COVID-19 displayed a lower risk of anxiety and depression. This might be due to the lack of clear and consistent guidelines on how to avoid and manage infections [81]. As reported, suicide/suicidal behaviors occurred not only among hospitalized patients, but also among uninfected people [82–84]. When facing a new outbreak, fear increases among the general population.

This study has a few limitations. Mental health and sexual dysfunction symptoms were assessed by questionnaires but not clinical diagnosis. Owing to the cross-sectional design, no causal associations could be derived from the study. Further studies are required to verify the potential long-term mental health problems associated with this pandemic.

## Conclusion

During the COVID-19 pandemic, anxiety, depression, and stress were moderately prevalent among infertile men in China. Combined with sexual dysfunction, drug therapy is closely associated with negative psychological outcomes. Risk factors also include sleep disorder symptoms, work being affected by outbreaks, and occupational exposure. Our findings suggest that the COVID-19 pandemic could have certain mental health repercussions on infertile men. We suggest that andrologists pay attention to mental health symptoms together with sexual function evaluations when treating infertile men. Psychological counseling and interpretation will also be useful.

## Supplementary Information

**Additional file 1: Fig 1.** The sampling process of the units that recruited infertile men.

**Additional file 2: Table 3.** Descriptive Statistics of Sexual function and COVID-19 Related Information for the Total Sample.

**Additional file 3:** **Table 2.** Demographic information for the Total Sample.

**Additional file 4: Table 1.** Summary of First Three Parts of the Questionnaire.

## Data Availability

The datasets used and analysed during the current study are available from the corresponding author on reasonable request.

## Abbreviations

COVID-19: coronavirus disease 2019
IIEF-5: International Index of Erectile Function-5 items
PEDT: Premature Ejaculation Diagnostic Tool
GAD-7: Generalized Anxiety Disorder-7
PHQ-9: Patient Health Questionnaire-9
IES-R: Impact of Event Scale-Revised
ED: erectile dysfunction
PE: premature ejaculation
ORs: odds ratios
CI: confidence interval
