# Supplementary material for: Psychological status of infertile men during the Coronavirus Disease 2019 Pandemic in China: a cross-sectional investigation
Source: Basic Clin Androl. 2023 Feb 16;33:8. doi: 10.1186/s12610-022-00177-5 (PMC9931448; doi:10.1186/s12610-022-00177-5)
Supplement: Supplementary file 1 — Additional file 1: Fig 1. The sampling process of the units that recruited infertile men. [file 12610_2022_177_MOESM1_ESM.docx]

**Psychological Status of Infertile Men during the Coronavirus Disease 2019 Pandemic in China: A Cross-sectional Investigation**

Zhe Zhang, PhD^1,2^*; Yu-Zhuo Yang, MD^2^*; Hai-Tao Zhang, PhD^1,2^*; Yu Xi, PhD^1,2^; Cun-Tong Wang, MD^3^; De-Feng Liu, MD^2^; Jia-Ming Mao, MD^2^; Hao-Cheng Lin, PhD^1,2^; Wen-Hao Tang, PhD^1,2^; Lian-Ming Zhao, PhD^1,2^; Xian-Sheng Zhang, PhD^4^; Yu-Tian Dai, PhD^5^; Hui Jiang, PhD^1,2^.

^1^ Department of Urology, Peking University Third Hospital, Beijing, China

^2^ Department of Reproductive Medicine Center, Peking University Third Hospital, Beijing, China

^3^ School of Social Development, Central University of Finance and Economics, Beijing, China

^4^ Department of Urology, The First Affiliated Hospital of Anhui Medical University, Hefei, China

^5^ Department of Andrology, Nanjing Drum Tower Hospital, Nanjing, China

*Contributed equally

**Correspondence:**

*Hui Jiang.* Department of Urology, Peking University Third Hospital, 49 Huayuan Rd, Beijing 100191, China.

Email address: jianghui@bjmu.edu.cn

*Yu-Tian Dai.* Department of Andrology, Nanjing Drum Tower Hospital, 321 Zhongshan Rd, Nanjing 210008, China.

Email address: yutian_dai@nju.edu.cn

*Xian-Sheng Zhang.* Department of Urology, The First Affiliated Hospital of Anhui Medical University, 218 Jixi Rd, Hefei 230022, China.

Email address: zhangxiansheng@ahmu.edu.cn

**Abstract**

**Background:** The coronavirus disease 2019 (COVID-19) outbreak has had a widespread and profound impact on people’s mental health. The factors associated with mental symptoms among men diagnosed with infertility, a disease closely related to psychological conditions, remain unclear. The aim of this study is to investigate the risk factors associated with mental symptoms among infertile Chinese men during the pandemic.

**Results:** A total of 4,098 eligible participants were recruited in this cross-sectional, nationwide study, including 2,034 (49.6%) with primary infertility and 2,064 (50.4%) with secondary infertility. The prevalence of mental health conditions was 36.3%, 39.6 %, and 6.7% for anxiety, depression, and post-pandemic stress, respectively. Sexual dysfunction is associated with a higher risk with adjusted odds ratios (ORs) of 1.40 for anxiety, 1.38 for depression, and 2.32 for stress. Men receiving infertility drug therapy displayed a higher risk for anxiety (adjusted OR, 1.31) and depression (adjusted OR, 1.28) symptoms, while those receiving intrauterine insemination had a lower risk of anxiety (adjusted OR, 0.56) and depression (adjusted OR, 0.55) symptoms.

**Conclusions:** The COVID-19 pandemic has had a significant psychological impact on infertile men. Several psychologically vulnerable populations were identified, including individuals with sexual dysfunction, respondents receiving infertility drug therapy, and those experiencing control measures for COVID-19. The findings provide a comprehensive profile of the mental health status of infertile Chinese men during the COVID-19 outbreak and provide potential psychological intervention strategies.

**Keywords:**

COVID-19, Male infertility, Psychological health, Sexual function

**Background**

The coronavirus disease 2019 (COVID-19) outbreak occurred in December 2019 and arousedhas attracted global attention [1]. According to WHO, which declared COVID-19 as a pandemic, the disease has infected more than 318 million people and caused more than six million deaths worldwide across more than 200 countries, areas, or territories as of October 2022 [2]. The daily life of most was significantly changed owing to the pandemic control measures. A series of measures were enforced in China, including restrictions on transport, entertainment, and social distancing measures [3-5]. Studies have shown that during a global pandemic, individuals may experience psychological problems [6] such as anxiety, depression, posttraumatic stress disorder, and negative societal behaviors such as societal rejection, discrimination, and stigmatization [7]. COVID-19 has had a serious impact on psychological and sexual health, as well as interpersonal relationships among the general population [6], especially in patients with stroke [8], hypertension [9], and Parkinson’s disease [10].

Infertility refers to the incapacity to conceive after one year of regular, unprotected sexual intercourse, and affects approximately one in five couples worldwide [11-13]. Male factors contribute to almost half of all cases, and approximately 7% of men worldwide are afflicted with this condition [14]. Indeed, it has been widely documented that being infertile significantly impacts the psychological well-being of both partners, who experience problems such as low self-esteem, sexual distress, depression, guilt, anxiety, frustration, and relational issues between themselves. Additionally, several studies have reported that these negative emotions can also be detrimental to sexual health and relationship quality, even conception, creating a vicious cycle [15]. For men, potential inverse dose-response relationships were revealed between anxiety [16] and depression [17] with sperm quality (concentration, motility, and total sperm count). Mental health symptoms are closely related to male sexual dysfunction [18-20], which is an important cause of male infertility. Infertility treatment is closely related to a patient's psychological condition, and dealing with infertility has a significant impact on their well-being and life satisfaction. These may originate from two underlying causes: fertility is related to patients’ interpersonal (social, relational, and marital) experiences, and it is connected to the importance of parenthood in a couple’s life [21-23].

The COVID-19 outbreak has had a widespread and profound impact on people’s psychological status, leading to the emergence of new mental symptoms or deterioration of existing mental illnesses [24-26]. Simultaneously, ongoing infertility treatment and medical support may be interrupted and delayed [27-29]. Infertile couples may feel isolated and neglected during this outbreak period, and may withdraw from their family and friends. A few studies have explored the impact of the pandemic on the mental health of female patients with infertility, and participants reported more negative emotions, including stress, worry, and frustration [27, 30, 31]. Infertile men can also experience a heavy psychological burden, especially under pressure from Chinese tradition and social role identification. Thus, to provide evidence-based guidance and help for infertile men during the COVID-19 pandemic, we conducted a large-sample cross-sectional study to investigate the mental health condition of Chinese infertile men.

**Methods**

***Study Design***

This cross-sectional study was conducted from September 4–21, 2020. A stratified cluster random sampling method was employed to recruit participants (Supplementary Figure 1), and the sample was obtained from the sampling frame of seven categories developed by stratifying all geographical regions. Sampling followed a tiered process that included three levels: provincial-level administrative regions, cities, and hospitals. Two representative provinces were randomly selected in each of the central and eastern regions (considering population density): one representative province was randomly selected in each of the other geographical regions, and the provincial capital city, together with two random secondary cities, were chosen in these provinces. Four tertiary hospitals and four secondary hospitals (two in the provincial capital city and one in each secondary city) were selected. Next, excessive sampling was conducted in Beijing, Shanghai, and Guangdong provinces, considering the economic level, urban size, and population density. Four tertiary and four secondary hospitals were selected from the three provincial-level administrative regions. Outpatient physicians involved in patient recruitment at all hospitals were trained to improve data collection consistency. Couples who had not been able to conceive a child even though they had frequent, unprotected sexual intercourse for a year or longer were recruited in the urology/andrology department and reproductive center. The husband’s personal, sexual, and medical/medication history were documented, after which he underwent a physical examination, semen analysis, and hormonal evaluation. Men with sperm disorders, hypogonadism, ejaculation disorders, or other factors affecting infertility were included in this study. Men with mental retardation or other diseases leading to an inability to understand the questionnaire items were excluded from this study. Written informed consent was obtained before the respondents completed the questionnaire.

***Questionnaires***

The survey comprised four parts. The first part gathered the demographic information of the participants, including age, education level, occupation, chronic disease and psychiatric disorder history, and sleeping status. The second part inquired about infertility status. The third part investigated pandemic-related conditions and attitudes toward the COVID-19 pandemic; we inquired about the COVID-19 infection history of participants and/or people around them, quarantine experiences, being frontline workers, and attitudes about COVID-19 affecting male reproduction. Information on these three parts is listed in Supplementary Table 1.

The fourth part of the questionnaire comprised five standardized scales, including the Chinese versions of the International Index of Erectile Function-5 items (IIEF-5) [32], Premature Ejaculation Diagnostic Tool (PEDT) [33], Generalized Anxiety Disorder-7 (GAD-7) [34], Patient Health Questionnaire-9 (PHQ-9) [35], and Impact of Event Scale-Revised (IES-R) [36], which measure symptoms of erectile dysfunction (ED), premature ejaculation (PE), anxiety, depression, and post-pandemic stress. The total scores of these scales were interpreted as follows: IIEF-5, normal (22–25), mild (17–21), and severe to moderate (5–16) erectile dysfunction (ED); PEDT, normal (0–8), suspicious (9–10), and confirmed (11–20) premature ejaculation (PE); GAD-7, normal (0–4), mild (5–9), moderate (10–14), and severe (15–21) anxiety; PHQ-9, normal (0–4), mild (5–9), moderate (10–14), and severe (15–21) depression; IES-R, normal (0–33); confirmed (34–88). In this study, cut-off scores of 5 for the GAD-7, 5 for the PHQ-9, and 34 for the IES-R were adopted to detect symptoms of anxiety, depression, and post-pandemic stress.

***Statistical Analysis***

Continuous variables are presented as means and standard deviations. Categorical variables are represented as absolute and percentage frequencies. All analyses were conducted using SPSS 20.0 (SPSS Inc., Chicago, IL, USA). All continuous variables were evaluated for normal distribution using the Shapiro-Wilk test; non-continuous variables with non-normal distribution of variance were evaluated using the Mann-Whitney U-test. Comparison of proportions was evaluated using the chi-square test. The Bonferroni method was used to adjust *P*-values when comparing the proportions. To explore factors potentially associated with anxiety, depression, and stress, unadjusted logistic regression and multivariable logistic regression analyses were performed. Odds ratios (ORs) and 95% confidence intervals (CIs) are presented. All statistical tests were two-tailed, and the significance level was set at *P* < 0.05.

**Results**

***Demographic information***

A total of 4,450 men were recruited from 96 urology/andrology and reproductive centers. After screening the incomplete questionnaires, data from 4,098 eligible participants were included in the final analysis, including 2,034 (49.6%) with primary infertility and 2,064 (50.4%) with secondary infertility. Of the total sample, 1,988 (48.5%) had been diagnosed with infertility for less than three years, 1,289 (31.5%) had been diagnosed for three years or more, and 821 (20.0%) had an uncertain infertility history. The mean age was 32.65±6.32 years old; 2,629 (64.2%) respondents possessed a college degree or higher; and 2,687 (65.6%) lived in urban areas. A total of 60.4% (2,476 cases) lived in a nuclear family and 80.3% (3,289 cases) held a steady job. More than half of the participants (2,150 men, 52.5%) had an average of more than eight hours of sleep per night during the pandemic, and 834 participants (20.4%) reported at least one sleeping disorder. Infertile men with ED and PE—the most common sexual dysfunction disorders—accounted for 57.1% and 15% of men, respectively. This survey included data from 61 individuals (1.5%) with confirmed or suspected cases of COVID-19, 30 (0.7%) individuals had been in close contact with COVID-19 patients, and 504 (12.3%) were frontline workers. A total of 793 (19.3%) participants underwent quarantine, and 2,705 (66.0%) reported that their daily work was affected by COVID-19. Additional demographic and pandemic-related characteristics are presented in Supplementary Tables 2 and 3.

***Prevalence of*** ***anxiety, depression, and*** ***post-pandemic stress symptoms***

In this study, 1,884 men (46.0%) had at least one psychological symptom, 1,011 (24.7%) had two different symptoms, and 244 (6.0%) had three different symptoms. The prevalence of symptoms for psychological conditions among infertile men was 36.3% (95% CI, 34.8–37.7%) for anxiety (1,486 participants total, including 1,139 participants [27.8%] with mild anxiety and 347 participants [8.5%] with moderate-to-severe anxiety), 39.6% (95% CI, 38.1–41.1%) for depression (1,623 participants total, including 1,149 participants [28.0%] with mild depression and 474 participants [11.6%] with moderate-to-severe depression), and 6.7% (95% CI, 5.9–7.5%) for post-pandemic stress (274 participants). The prevalence of anxiety (39.3%), depression (43.8%), and post-pandemic stress symptoms (7.9%) in eastern China was significantly higher than that in other areas of China, while depression and post-pandemic stress were significantly lower in northern China (35.4% and 3.3%, respectively). The prevalence of anxiety, depression, and post-pandemic stress symptoms in the different geographical regions is shown in Figure 2.

Among 1,486 men with anxiety symptoms, 1,021 (68.7%) had ED symptoms and 495 (33.3%) had PE symptoms. In those with depressive symptoms (1,623 men), 1,125 (69.3%) had ED symptoms and 549 (33.8%) had PE symptoms. The percentage of participants with post-pandemic stress symptoms increased to 78.8% (ED, 216 men) and 48.9% (PE, 134 men). Of the 994 men with moderate to severe ED symptoms, 146 (14.7%) had moderate to severe anxiety symptoms, 209 (21.0%) had moderate to severe depressive symptoms, and 132 (13.3%) had stress symptoms, as measured by the validated questionnaires. Among the 613 men with PE symptoms, 116 (18.9%) had moderate to severe anxiety symptoms, 157 (25.6%) had moderate to severe depressive symptoms, and 92 (15.0%) had stress symptoms. The prevalence of psychological symptoms was high among men with insomnia (anxiety, 66.5%; depression, 72.7%; stress, 19.8%), sleep duration less than eight hours (anxiety, 41.7%; depression, 45.1%; stress, 8.0%), men on medication (anxiety, 42.9%; depression, 46.0%; stress, 7.6%), frontline workers (anxiety, 42.1%; depression, 48.6%), participants who experienced both centralized quarantine (anxiety, 42.9%; depression, 52.4%) and home quarantine (anxiety, 42.6%; depression, 48.6%), participants with working delayed (anxiety, 38.8%; depression, 43.6%; stress, 7.4%), participants out of work (anxiety, 36.5%; depression, 39.9%; stress, 12.2%), participants experiencing salary cuts or unemployment during COVID-19 (anxiety, 43.4%; depression, 47.1%; stress, 8.5%), and participants reporting increase in workload (anxiety, 43.7%; depression, 47.3%; stress, 9.0%). Meanwhile, the prevalence of psychological symptoms was also high among men with the following conditions: fever, fatigue, or headache (anxiety, 53.1%; depression, 70.3%; stress, 14.1%), worrying about being infected (anxiety, 44.2%; depression, 48.4%; stress, 9.9%), being concerned about the impact of COVID-19 on sexual function (anxiety, 49.5%; depression, 53.3%; stress, 15.7%), and choosing cryopreservation of sperm in response to COVID-19 (anxiety, 44.4%; depression, 44.6%; stress, 13.2%). The prevalence of psychological problems among the different populations is presented in Table 1.

***Factors associated with symptoms of anxiety, depression, and post-pandemic stress***

In the multivariable analysis, participants with a college or higher educational background and history of psychiatric disorders displayed a remarkably higher risk of anxiety and depression symptoms. Sleeping disorders, ED, and PE were found to be associated with symptoms of anxiety, depression, and stress, and participants with sleep duration longer than eight hours had a lower risk of anxiety (adjusted OR, 0.84; 95% CI, 0.73–0.97) and depression (adjusted OR, 0.86; 95% CI, 0.74–1.00) symptoms. Simultaneously, being concerned about the impact of COVID-19 on sexual function is associated with a higher risk with adjusted ORs of 1.40 (95% CI, 1.18–1.67) for anxiety, 1.38 (95% CI, 1.16–1.64) for depression, and 2.29 (95% CI, 1.70–3.08) for post-pandemic stress symptoms. Men receiving infertility drug therapy displayed a higher risk of anxiety (adjusted OR, 1.30; 95% CI, 1.12–1.52) and depression (adjusted OR, 1.27; 95% CI, 1.09–1.48) symptoms. Men considering cryopreservation of sperm in response to the pandemic had an elevated risk of stress symptoms (adjusted OR, 1.50; 95% CI, 1.06–2.10). Participants whose work was affected by COVID-19 faced higher risks than those whose work was not affected. Among these impacts, salary cut/job loss was associated with at least twice the risk of anxiety, depression, and stress symptoms (adjusted ORs, 2.02 [95% CI, 1.70–2.41] for anxiety, 2.16 [95% CI, 1.81–2.57] for depression, and 2.01 [95% CI, 1.39–2.89] for stress symptoms). Men out of work were highly susceptible to symptoms of stress (adjusted OR, 3.64; 95% CI, 1.98–6.70). Men worried about COVID-19 infection demonstrated a higher risk with adjusted ORs of 1.41 (95% CI, 1.22–1.64) for anxiety, 1.33 (95% CI, 1.13–1.55) for depression, and 1.53 (95% CI, 1.15–2.03) for stress symptoms. Infertile men with a higher desire to receive psychological counseling during the pandemic demonstrated a higher risk of anxiety (adjusted OR, 1.10; 95% CI, 1.08–1.13), depression (adjusted OR, 1.09; 95% CI, 1.06–1.11), and stress (adjusted OR, 1.15; 95% CI, 1.11–1.20). Nonetheless, men with more knowledge about COVID-19 displayed a lower risk of anxiety (adjusted OR, 0.94; 95% CI, 0.91–0.98) and depression (adjusted OR, 0.95; 95% CI, 0.91–0.98). Other risk factors for depressive symptoms included fever, fatigue, or headache during the pandemic (adjusted OR, 2.26; 95% CI, 1.22–4.21), being frontline workers (adjusted OR, 1.36; 95% CI, 1.09–1.70), and home quarantine experiences (adjusted OR, 1.29; 95% CI, 1.08–1.55). Detailed results of the multivariable analysis are presented in Table 2.

**Discussion**

Severe emotional distress can occur during public health events [37-40]. To the best of our knowledge, numerous studies have examined women with infertility (Table 3). Ceasing infertility treatment was the primary problem affecting patients [27, 42, 43], while changes in working environment and style [44], quarantine [45], and financial concerns [46] increase the psychological burden of patients. Negative emotions were found to reduce the quality of couples’ relationships [28] and lower expectations of future pregnancy [47] during the pandemic. Men with infertility are especially vulnerable to psychological problems [41], and the psychological status of infertile male patients is often overlooked. This is the first nationwide study of an infertile male population that systematically investigated the prevalence of and factors associated with mental health symptoms using standardized rating scales during this COVID-19 outbreak period. This study revealed that the pandemic has had a psychological impact on infertile men, and approximately half of them had at least one psychological symptom. More than one-third of infertile men developed anxiety or depression symptoms, and a certain percentage had post-pandemic stress symptoms. Several psychologically vulnerable populations were identified, including individuals with sexual dysfunction, respondents receiving infertility drug therapy, those with work being affected, those who experienced home quarantine, and frontline workers. The findings provide a comprehensive profile of the mental health status of infertile Chinese men during the COVID-19 outbreak and provide potential psychological intervention strategies.

The prevalence of anxiety was consistent and depression was higher compared to studies of Chinese infertile men before the COVID-19 outbreak by Gao et al. [48] in 2013 and Ma et al. [20] in 2017. Meanwhile, we found that the prevalence of psychological symptoms was also higher compared to data from the general population during the COVID-19 pandemic [26, 49], which was reported to be approximately one-fifth to one-third. This indicates that the pandemic’s psychological impact is greater on infertile men than on the general population.

As our data showed, several groups were likely to develop psychological symptoms. This study revealed that erection/ejaculation dysfunction leads to a higher risk of mental health symptoms such as anxiety, depression, and stress. PE and ED are two major male sexual dysfunctions with prevalence varying from 20% to 50% in different districts, and the prevalence is even higher among infertile populations [50-54]. The prevalence of ED was reported to increase due to severe semen quality impairment [55]. In daily clinical practice, approximately 50–80% of men with sexual dysfunction show concomitant symptoms of depression or anxiety [18]. Poor mental health can also lead to ED or PE [56-58]. Ejaculatory latency, sexual desire, and orgasmic function are reduced in infertile men and are mainly associated with mood disturbances [55]. The frequency of sexual intercourse was significantly related to changes in erectile function and ejaculatory control ability, and participants with a low frequency of sexual intercourse had less partner time and intimate behavior with sexual partners. Owing to the restrictions on social activity and transport during the pandemic, partners who did not live together had a lower chance of sexual intercourse [59-62]. As two entities that cause and affect each other, sexual function and psychological status must be assessed in infertile populations. A deep analysis of the sexual and psychological status of infertile men, as well as evaluating their association, might be helpful in exploring the effects of infertility on sexual function and possible treatment plans [48], and in improving not only reproductive but also general and sexual health [55].

Drug therapy is usually the first choice in idiopathic male infertility and in men with an abnormality in the hypothalamic-pituitary-testicular axis, leading to endocrine-related infertility [11]. Although drug treatment is a non-invasive and affordable choice, patients still have to pin their hopes on the efficacy of drugs and wait to conceive naturally. Considering the spermatogenesis cycle, the duration of drug treatment is usually 6–12 months, at least [63]. During this pandemic, fertility treatment suspensions have had a considerable negative impact on patients’ mental health and quality of life [42]. Men on medication are likely to experience deterioration of their psychological condition due to a lack of further medical attention.

The secondary social impacts of COVID-19 were identified as boosters for psychological symptoms in this study, which is consistent with a UK-based finding that individuals were more concerned about the secondary social impacts of COVID-19 than the direct threats of COVID-19 infection [64]. Infertile men whose work was affected reported a higher risk for symptoms of anxiety, depression, and post-pandemic stress. As reported in Australia, financial pressure due to salary cut/job loss, which was also commonly identified in our sample, was associated with poorer mental health [65]. Our study also identified home quarantine experience as a risk factor for depressive symptoms, in line with study results from the general population [49, 66]. Owing to the strict policy on social contact, procedures in various industries have slowed down and inevitably leading to delays/backlogs. The lack of office connections might reduce creativity and productivity and is not conducive to the discharge of mental pressure. In China, men are usually the family breadwinners. The negative impact of COVID-19 on work will worsen the situation for men struggling with fertility problems, giving rise to psychological symptoms.

We found that the threat of COVID-19 infection could also contribute to psychological symptoms. A high probability of depressive symptoms was found among frontline workers. People infected with the coronavirus may be asymptomatic during the incubation period, and its clinical manifestations can be easily confused with those of normal influenza [67-69]. During this public health emergency, frontline workers may fear getting sick and spreading the infection to their families, other patients, and coworkers [70]. As our data suggested, men worrying about themselves or their relatives being infected with COVID-19 showed a higher risk of anxiety, depression, and stress, and men with fever, fatigue, or headaches during the pandemic were more vulnerable to depression symptoms. It has been suggested that the coronavirus infection could impair the male reproductive system if spermatogenic tubules, testicular stromal cells, and spermatogenic cells in the testis are invaded by the virus [71-74]. Testicular damage was found to be more severe in men of procreant age than in older men [75-77]. This implies that the potential after-effects of even mild infections are magnified in infertile men and put them under substantial psychological burden. This was further confirmed by our finding that men choosing cryopreservation of sperm in response to the COVID-19 pandemic had a higher risk of stress symptoms.

Sleep conditions may also have influenced mental health during the pandemic. Self-reported insomnia and snoring are the risk factors for anxiety, depression, and stress. The unsettling news and images shown on television and the Internet, together with the uncertainty of the novel virus, might have contributed to the emergence of sleep disorders. Our results demonstrated that snoring can affect both sleep duration and the quality of sleep [78]. Sleep disorders are closely related to anxiety, depression, and stress [79, 80]. Infertile men who claimed to know about COVID-19 displayed a lower risk of anxiety and depression. This might be due to the lack of clear and consistent guidelines on how to avoid and manage infections [81]. As reported, suicide/suicidal behaviors occurred not only among hospitalized patients, but also among uninfected people [82-84]. When facing a new outbreak, fear increases among the general population.

This study has a few limitations. Mental health and sexual dysfunction symptoms were assessed by questionnaires but not clinical diagnosis. Owing to the cross-sectional design, no causal associations could be derived from the study. Further studies are required to verify the potential long-term mental health problems associated with this pandemic.

**Conclusions**

During the COVID-19 pandemic, anxiety, depression, and stress were moderately prevalent among infertile men in China. Combined with sexual dysfunction, drug therapy is closely associated with negative psychological outcomes. Risk factors also include sleep disorder symptoms, work being affected by outbreaks, and occupational exposure. Our findings suggest that the COVID-19 pandemic could have certain mental health repercussions on infertile men. We suggest that andrologists pay attention to mental health symptoms together with sexual function evaluations when treating infertile men. Psychological counseling and interpretation will also be useful.

**List of abbreviations**

COVID-19: coronavirus disease 2019

IIEF-5: International Index of Erectile Function-5 items

PEDT: Premature Ejaculation Diagnostic Tool

GAD-7: Generalized Anxiety Disorder-7

PHQ-9: Patient Health Questionnaire-9

IES-R: Impact of Event Scale-Revised

ED: erectile dysfunction

PE: premature ejaculation

ORs: odds ratios

CI: confidence interval

**Declarations**

***Ethics approval and consent to participate***

The study was approved by the ethics committee of Peking University Third Hospital (Ethics Registration Number 2020SZ035).

***Consent for publication***

All authors contributed to the study. They have all seen the final version and consent to its submission.

***Availability of data and materials***

The datasets used and analysed during the current study are available from the corresponding author on reasonable request.

***Competing interests***

The authors declare that they have no competing interests.

***Funding***

This study was supported by grant 2021YFC2700203 from the National Key Research & Developmental Program of China, grant 7222208 from the Natural Science Foundation of Beijing Municipality, and grants 81901535 and 82071698 from National Natural Science Foundation of China.

***Authors' contributions***

Conceptualization, Z.Z. and H.J.; Methodology, Z.Z. and C.W.; Validation, Y.X., Y.Y, and H.Z.; Formal Analysis, Y.X. and Z.Z.; Investigation, Z.Z., X.Z. and Y.D.; Resources, D.L., J.M., H.L., W.T., L.Z., and H.J.; Data Curation, Z.Z.; Writing – Original Draft, Y.Y., H.Z., Y.X. and Z.Z.; Writing – Review & Editing, Y.Y., H.Z., Z.Z., and H.J.; Visualization, Y.X.; Supervision, Z.Z., X.Z., Y.D., and H.J.; Project Administration, Z.Z. and H.J.

***Acknowledgements***

Not applicable.

**References**

1. Wang C, Horby PW, Hayden FG, Gao GF. A novel coronavirus outbreak of global health concern. Lancet. 2020;395(10223):470-3. doi: 10.1016/S0140-6736(20)30185-9

2. World Health Organization. Coronavirus disease (COVID-19) pandemic. 2021 [Available from: <https://www.who.int/emergencies/diseases/novel-coronavirus-2019>.

3. Colbourn T. COVID-19: extending or relaxing distancing control measures. Lancet Public Health. 2020;5(5):e236-e7. doi: 10.1016/S2468-2667(20)30072-4

4. Koo JR, Cook AR, Park M, Sun Y, Sun H, Lim JT, et al. Interventions to mitigate early spread of SARS-CoV-2 in Singapore: a modelling study. Lancet Infect Dis. 2020;20(6):678-88. doi: 10.1016/S1473-3099(20)30162-6

5. Prem K, Liu Y, Russell TW, Kucharski AJ, Eggo RM, Davies N, et al. The effect of control strategies to reduce social mixing on outcomes of the COVID-19 epidemic in Wuhan, China: a modelling study. Lancet Public Health. 2020;5(5):e261-e70. doi: 10.1016/S2468-2667(20)30073-6

6. Cosic K, Popovic S, Sarlija M, Kesedzic I. Impact of Human Disasters and COVID-19 Pandemic on Mental Health: Potential of Digital Psychiatry. Psychiatr Danub. 2020;32(1):25-31. doi: 10.24869/psyd.2020.25

7. Shigemura J, Ursano RJ, Morganstein JC, Kurosawa M, Benedek DM. Public responses to the novel 2019 coronavirus (2019-nCoV) in Japan: Mental health consequences and target populations. Psychiatry Clin Neurosci. 2020;74(4):281-2. doi: 10.1111/pcn.12988

8. Chou HY, Lo YC, Tsai YW, Shih CL, Yeh CT. Increased Anxiety and Depression Symptoms in Post-Acute Care Patients with Stroke during the COVID-19 Pandemic. Int J Environ Res Public Health. 2021;19(1). doi: 10.3390/ijerph19010162

9. Dobler CL, Kruger B, Strahler J, Weyh C, Gebhardt K, Tello K, et al. Physical Activity and Mental Health of Patients with Pulmonary Hypertension during the COVID-19 Pandemic. J Clin Med. 2020;9(12). doi: 10.3390/jcm9124023

10. Shalash A, Roushdy T, Essam M, Fathy M, Dawood NL, Abushady EM, et al. Mental Health, Physical Activity, and Quality of Life in Parkinson's Disease During COVID-19 Pandemic. Mov Disord. 2020;35(7):1097-9. doi: 10.1002/mds.28134

11. Agarwal A, Mulgund A, Hamada A, Chyatte MR. A unique view on male infertility around the globe. Reprod Biol Endocrinol. 2015;13:37. doi: 10.1186/s12958-015-0032-1

12. Dimitriadis F, Tsounapi P, Zachariou A, Kaltsas A, Sokolakis I, Hatzichristodoulou G, et al. Therapeutic Effects of Micronutrient Supplements on Sperm Parameters: Fact or Fiction? Curr Pharm Des. 2021;27(24):2757-69. doi: 10.2174/1381612826666200415173537

13. Kopa Z, Keszthelyi M, Sofikitis N. Administration of Antioxidants in the Infertile Male: When it may have a Beneficial Effect? Curr Pharm Des. 2021;27(23):2665-8. doi: 10.2174/1381612826666200303115552

14. Agarwal A, Bui AD. Oxidation-reduction potential as a new marker for oxidative stress: Correlation to male infertility. Investig Clin Urol. 2017;58(6):385-99. doi: 10.4111/icu.2017.58.6.385

15. La Rosa VL, Barra F, Chiofalo B, Platania A, Di Guardo F, Conway F, et al. An overview on the relationship between endometriosis and infertility: the impact on sexuality and psychological well-being. J Psychosom Obstet Gynaecol. 2020;41(2):93-7. doi: 10.1080/0167482X.2019.1659775

16. Peaston G, Subramanian V, Brunckhorst O, Sarris I, Ahmed K. The impact of emotional health on assisted reproductive technology outcomes: a systematic review and meta-analysis. Hum Fertil (Camb). 2020:1-12. doi: 10.1080/14647273.2020.1832262

17. Ye YX, Chen HG, Sun B, Chen YJ, Duan P, Meng TQ, et al. Associations between depression, oxidative stress, and semen quality among 1,000 healthy men screened as potential sperm donors. Fertil Steril. 2022;117(1):86-94. doi: 10.1016/j.fertnstert.2021.09.013

18. Yang Y, Song Y, Lu Y, Xu Y, Liu L, Liu X. Associations between erectile dysfunction and psychological disorders (depression and anxiety): A cross-sectional study in a Chinese population. Andrologia. 2019;51(10):e13395. doi: 10.1111/and.13395

19. Lu Y, Fan S, Cui J, Yang Y, Song Y, Kang J, et al. The decline in sexual function, psychological disorders (anxiety and depression) and life satisfaction in older men: A cross-sectional study in a hospital-based population. Andrologia. 2020;52(5):e13559. doi: 10.1111/and.13559

20. Ma J, Wang B, Dang J, Li X, Ding J, Zhu Y, et al. Erectile dysfunction and psychological status in infertile males. National Journal of Andrology. 2017;23(07):609-14. doi: 10.13263/j.cnki.nja.2017.07.006

21. Donarelli Z, Gullo S, Lo Coco G, Marino A, Scaglione P, Volpes A, et al. Assessing infertility-related stress: the factor structure of the Fertility Problem Inventory in Italian couples undergoing infertility treatment. J Psychosom Obstet Gynaecol. 2015;36(2):58-65. doi: 10.3109/0167482X.2015.1034268

22. Donarelli Z, Lo Coco G, Gullo S, Salerno L, Marino A, Sammartano F, et al. The Fertility Quality of Life Questionnaire (FertiQoL) Relational subscale: psychometric properties and discriminant validity across gender. Hum Reprod. 2016;31(9):2061-71. doi: 10.1093/humrep/dew168

23. Moura-Ramos M, Gameiro S, Canavarro MC, Soares I. Assessing infertility stress: re-examining the factor structure of the Fertility Problem Inventory. Hum Reprod. 2012;27(2):496-505. doi: 10.1093/humrep/der388

24. Hall RC, Hall RC, Chapman MJ. The 1995 Kikwit Ebola outbreak: lessons hospitals and physicians can apply to future viral epidemics. Gen Hosp Psychiatry. 2008;30(5):446-52. doi: 10.1016/j.genhosppsych.2008.05.003

25. Bao Y, Sun Y, Meng S, Shi J, Lu L. 2019-nCoV epidemic: address mental health care to empower society. Lancet. 2020;395(10224):e37-e8. doi: 10.1016/S0140-6736(20)30309-3

26. Wang C, Pan R, Wan X, Tan Y, Xu L, Ho CS, et al. Immediate Psychological Responses and Associated Factors during the Initial Stage of the 2019 Coronavirus Disease (COVID-19) Epidemic among the General Population in China. Int J Environ Res Public Health. 2020;17(5). doi: 10.3390/ijerph17051729

27. Lawson AK, McQueen DB, Swanson AC, Confino R, Feinberg EC, Pavone ME. Psychological distress and postponed fertility care during the COVID-19 pandemic. J Assist Reprod Genet. 2021;38(2):333-41. doi: 10.1007/s10815-020-02023-x

28. Dong M, Wu S, Tao Y, Zhou F, Tan J. The Impact of Postponed Fertility Treatment on the Sexual Health of Infertile Patients Owing to the COVID-19 Pandemic. Front Med (Lausanne). 2021;8:730994. doi: 10.3389/fmed.2021.730994

29. Barra F, La Rosa VL, Vitale SG, Commodari E, Altieri M, Scala C, et al. Psychological status of infertile patients who had in vitro fertilization treatment interrupted or postponed due to COVID-19 pandemic: a cross-sectional study. J Psychosom Obstet Gynaecol. 2020:1-8. doi: 10.1080/0167482X.2020.1853095

30. Ben-Kimhy R, Youngster M, Medina-Artom TR, Avraham S, Gat I, Marom Haham L, et al. Fertility patients under COVID-19: attitudes, perceptions and psychological reactions. Hum Reprod. 2020;35(12):2774-83. doi: 10.1093/humrep/deaa248

31. Boivin J, Harrison C, Mathur R, Burns G, Pericleous-Smith A, Gameiro S. Patient experiences of fertility clinic closure during the COVID-19 pandemic: appraisals, coping and emotions. Hum Reprod. 2020;35(11):2556-66. doi: 10.1093/humrep/deaa218

32. Huang YF, Li HJ. Practical andrology: Beijing: Science Press; 2009.

33. Huang YP, Chen B, Ping P, Wang HX, Hu K, Zhang T, et al. The premature ejaculation diagnostic tool (PEDT): linguistic validity of the Chinese version. J Sex Med. 2014;11(9):2232-8. doi: 10.1111/jsm.12612

34. He XY, Li C, Qian J, Cui HS, Wu WY. Reliability and validity of a generalized anxiety scale in general hospital outpatients. Shanghai Archives of Psychiatry. 2010;22:200-3. doi: 10.3969/j.issn.1002-0829.2010.04.002

35. Sun XY, Li YX, Yu CQ, Li LM. [Reliability and validity of depression scales of Chinese version: a systematic review]. Zhonghua Liu Xing Bing Xue Za Zhi. 2017;38(1):110-6. doi: 10.3760/cma.j.issn.0254-6450.2017.01.021

36. Wu KK, Chan KS. The development of the Chinese version of Impact of Event Scale--Revised (CIES-R). Soc Psychiatry Psychiatr Epidemiol. 2003;38(2):94-8. doi: 10.1007/s00127-003-0611-x

37. Sim K, Huak Chan Y, Chong PN, Chua HC, Wen Soon S. Psychosocial and coping responses within the community health care setting towards a national outbreak of an infectious disease. J Psychosom Res. 2010;68(2):195-202. doi: 10.1016/j.jpsychores.2009.04.004

38. Taylor MR, Agho KE, Stevens GJ, Raphael B. Factors influencing psychological distress during a disease epidemic: data from Australia's first outbreak of equine influenza. BMC Public Health. 2008;8:347. doi: 10.1186/1471-2458-8-347

39. Thienkrua W, Cardozo BL, Chakkraband ML, Guadamuz TE, Pengjuntr W, Tantipiwatanaskul P, et al. Symptoms of posttraumatic stress disorder and depression among children in tsunami-affected areas in southern Thailand. JAMA. 2006;296(5):549-59. doi: 10.1001/jama.296.5.549

40. van Griensven F, Chakkraband ML, Thienkrua W, Pengjuntr W, Lopes Cardozo B, Tantipiwatanaskul P, et al. Mental health problems among adults in tsunami-affected areas in southern Thailand. JAMA. 2006;296(5):537-48. doi: 10.1001/jama.296.5.537

41. Esteves SC, Lombardo F, Garrido N, Alvarez J, Zini A, Colpi GM, et al. SARS-CoV-2 pandemic and repercussions for male infertility patients: A proposal for the individualized provision of andrological services. Andrology. 2021;9(1):10-8. doi: 10.1111/andr.12809

42. Gordon JL, Balsom AA. The psychological impact of fertility treatment suspensions during the COVID-19 pandemic. PLoS One. 2020;15(9):e0239253. doi: 10.1371/journal.pone.0239253

43. Tippett A. Life on pause: An analysis of UK fertility patients' coping mechanisms after the cancellation of fertility treatment due to COVID-19. J Health Psychol. 2022;27(7):1583-600. doi: 10.1177/1359105321999711

44. Galhardo A, Carolino N, Monteiro B, Cunha M. The emotional impact of the Covid-19 pandemic in women facing infertility. Psychol Health Med. 2022;27(2):389-95. doi: 10.1080/13548506.2021.1922721

45. Cao LB, Hao Q, Liu Y, Sun Q, Wu B, Chen L, et al. Anxiety Level During the Second Localized COVID-19 Pandemic Among Quarantined Infertile Women: A Cross-Sectional Survey in China. Front Psychiatry. 2021;12:647483. doi: 10.3389/fpsyt.2021.647483

46. Seifer DB, Petok WD, Agrawal A, Glenn TL, Bayer AH, Witt BR, et al. Psychological experience and coping strategies of patients in the Northeast US delaying care for infertility during the COVID-19 pandemic. Reprod Biol Endocrinol. 2021;19(1):28. doi: 10.1186/s12958-021-00721-4

47. Dillard AJ, Weber AE, Chassee A, Thakur M. Perceptions of the COVID-19 Pandemic among Women with Infertility: Correlations with Dispositional Optimism. Int J Environ Res Public Health. 2022;19(5). doi: 10.3390/ijerph19052577

48. Gao J, Zhang X, Su P, Liu J, Shi K, Hao Z, et al. Relationship between sexual dysfunction and psychological burden in men with infertility: a large observational study in China. J Sex Med. 2013;10(8):1935-42. doi: 10.1111/jsm.12207

49. Shi L, Lu ZA, Que JY, Huang XL, Liu L, Ran MS, et al. Prevalence of and Risk Factors Associated With Mental Health Symptoms Among the General Population in China During the Coronavirus Disease 2019 Pandemic. JAMA Netw Open. 2020;3(7):e2014053. doi: 10.1001/jamanetworkopen.2020.14053

50. Lotti F, Corona G, Rastrelli G, Forti G, Jannini EA, Maggi M. Clinical correlates of erectile dysfunction and premature ejaculation in men with couple infertility. J Sex Med. 2012;9(10):2698-707. doi: 10.1111/j.1743-6109.2012.02872.x

51. Jain K, Radhakrishnan G, Agrawal P. Infertility and psychosexual disorders: relationship in infertile couples. Indian J Med Sci. 2000;54(1):1-7. doi:

52. Shindel AW, Nelson CJ, Naughton CK, Mulhall JP. Premature ejaculation in infertile couples: prevalence and correlates. J Sex Med. 2008;5(2):485-91. doi: 10.1111/j.1743-6109.2007.00690.x

53. Hurwitz MB. Sexual dysfunction associated with infertility. A comparison of sexual function during the fertile and the non-fertile phase of the menstrual cycle. S Afr Med J. 1989;76(2):58-61. doi:

54. Shindel AW, Nelson CJ, Naughton CK, Ohebshalom M, Mulhall JP. Sexual function and quality of life in the male partner of infertile couples: prevalence and correlates of dysfunction. J Urol. 2008;179(3):1056-9. doi: 10.1016/j.juro.2007.10.069

55. Lotti F, Corona G, Castellini G, Maseroli E, Fino MG, Cozzolino M, et al. Semen quality impairment is associated with sexual dysfunction according to its severity. Hum Reprod. 2016;31(12):2668-80. doi: 10.1093/humrep/dew246

56. Starc A, Trampus M, Pavan Jukic D, Rotim C, Jukic T, Polona Mivsek A. Infertility and Sexual Dysfunctions: A Systematic Literature Review. Acta Clin Croat. 2019;58(3):508-15. doi: 10.20471/acc.2019.58.03.15

57. Berger MH, Messore M, Pastuszak AW, Ramasamy R. Association Between Infertility and Sexual Dysfunction in Men and Women. Sex Med Rev. 2016;4(4):353-65. doi: 10.1016/j.sxmr.2016.05.002

58. Lotti F, Maggi M. Sexual dysfunction and male infertility. Nat Rev Urol. 2018;15(5):287-307. doi: 10.1038/nrurol.2018.20

59. Cito G, Micelli E, Cocci A, Polloni G, Russo GI, Coccia ME, et al. The Impact of the COVID-19 Quarantine on Sexual Life in Italy. Urology. 2021;147:37-42. doi: 10.1016/j.urology.2020.06.101

60. Jacob L, Smith L, Butler L, Barnett Y, Grabovac I, McDermott D, et al. Challenges in the Practice of Sexual Medicine in the Time of COVID-19 in the United Kingdom. J Sex Med. 2020;17(7):1229-36. doi: 10.1016/j.jsxm.2020.05.001

61. Li W, Li G, Xin C, Wang Y, Yang S. Challenges in the Practice of Sexual Medicine in the Time of COVID-19 in China. J Sex Med. 2020;17(7):1225-8. doi: 10.1016/j.jsxm.2020.04.380

62. Li G, Tang D, Song B, Wang C, Qunshan S, Xu C, et al. Impact of the COVID-19 Pandemic on Partner Relationships and Sexual and Reproductive Health: Cross-Sectional, Online Survey Study. J Med Internet Res. 2020;22(8):e20961. doi: 10.2196/20961

63. Valenti D, La Vignera S, Condorelli RA, Rago R, Barone N, Vicari E, et al. Follicle-stimulating hormone treatment in normogonadotropic infertile men. Nat Rev Urol. 2013;10(1):55-62. doi: 10.1038/nrurol.2012.234

64. Holmes EA, O'Connor RC, Perry VH, Tracey I, Wessely S, Arseneault L, et al. Multidisciplinary research priorities for the COVID-19 pandemic: a call for action for mental health science. Lancet Psychiatry. 2020;7(6):547-60. doi: 10.1016/S2215-0366(20)30168-1

65. Dawel A, Shou Y, Smithson M, Cherbuin N, Banfield M, Calear AL, et al. The Effect of COVID-19 on Mental Health and Wellbeing in a Representative Sample of Australian Adults. Front Psychiatry. 2020;11:579985. doi: 10.3389/fpsyt.2020.579985

66. Brooks SK, Webster RK, Smith LE, Woodland L, Wessely S, Greenberg N, et al. The psychological impact of quarantine and how to reduce it: rapid review of the evidence. Lancet. 2020;395(10227):912-20. doi: 10.1016/S0140-6736(20)30460-8

67. Bai Y, Yao L, Wei T, Tian F, Jin DY, Chen L, et al. Presumed Asymptomatic Carrier Transmission of COVID-19. JAMA. 2020;323(14):1406-7. doi: 10.1001/jama.2020.2565

68. Chen N, Zhou M, Dong X, Qu J, Gong F, Han Y, et al. Epidemiological and clinical characteristics of 99 cases of 2019 novel coronavirus pneumonia in Wuhan, China: a descriptive study. Lancet. 2020;395(10223):507-13. doi: 10.1016/S0140-6736(20)30211-7

69. Godeau D, Petit A, Richard I, Roquelaure Y, Descatha A. Return-to-work, disabilities and occupational health in the age of COVID-19. Scand J Work Environ Health. 2021;47(5):408-9. doi: 10.5271/sjweh.3960

70. Thakur B, Pathak M. Burden of Predominant Psychological Reactions Among the Healthcare Workers and General Population During COVID-19 Pandemic Phase: A Systematic Review and Meta-Analysis. Indian J Community Med. 2021;46(4):600-5. doi: 10.4103/ijcm.IJCM_1007_20

71. Wang N, Qin L, Ma L, Yan H. Effect of severe acute respiratory syndrome coronavirus-2 (SARS-CoV-2) on reproductive system. Stem Cell Res. 2021;52:102189. doi: 10.1016/j.scr.2021.102189

72. Groner MF, de Carvalho RC, Camillo J, Ferreira PRA, Fraietta R. Effects of Covid-19 on male reproductive system. Int Braz J Urol. 2021;47(1):185-90. doi: 10.1590/S1677-5538.IBJU.2021.99.04

73. Wang Z, Xu X. scRNA-seq Profiling of Human Testes Reveals the Presence of the ACE2 Receptor, A Target for SARS-CoV-2 Infection in Spermatogonia, Leydig and Sertoli Cells. Cells. 2020;9(4). doi: 10.3390/cells9040920

74. Vaz-Silva J, Carneiro MM, Ferreira MC, Pinheiro SV, Silva DA, Silva-Filho AL, et al. The vasoactive peptide angiotensin-(1-7), its receptor Mas and the angiotensin-converting enzyme type 2 are expressed in the human endometrium. Reprod Sci. 2009;16(3):247-56. doi: 10.1177/1933719108327593

75. Holtmann N, Edimiris P, Andree M, Doehmen C, Baston-Buest D, Adams O, et al. Assessment of SARS-CoV-2 in human semen-a cohort study. Fertil Steril. 2020;114(2):233-8. doi: 10.1016/j.fertnstert.2020.05.028

76. Illiano E, Trama F, Costantini E. Could COVID-19 have an impact on male fertility? Andrologia. 2020;52(6):e13654. doi: 10.1111/and.13654

77. Pan F, Xiao X, Guo J, Song Y, Li H, Patel DP, et al. No evidence of severe acute respiratory syndrome-coronavirus 2 in semen of males recovering from coronavirus disease 2019. Fertil Steril. 2020;113(6):1135-9. doi: 10.1016/j.fertnstert.2020.04.024

78. Eisenberg E, Legro RS, Diamond MP, Huang H, O'Brien LM, Smith YR, et al. Sleep Habits of Women With Infertility. J Clin Endocrinol Metab. 2021;106(11):e4414-e26. doi: 10.1210/clinem/dgab474

79. Huang LH, Kuo CP, Lu YC, Lee MS, Lee SH. Association of emotional distress and quality of sleep among women receiving in-vitro fertilization treatment. Taiwan J Obstet Gynecol. 2019;58(1):168-72. doi: 10.1016/j.tjog.2018.11.031

80. Itoh Y, Takeshima M, Kaneita Y, Uchimura N, Inoue Y, Honda M, et al. Associations Between the 2011 Great East Japan Earthquake and Tsunami and the Sleep and Mental Health of Japanese People: A 3-Wave Repeated Survey. Nat Sci Sleep. 2022;14:61-73. doi: 10.2147/NSS.S338095

81. Patrucco F, Gavelli F, Fagoonee S, Solidoro P, Undas A, Pellicano R. Current treatment challenges in the COVID-19 pandemic. Pol Arch Intern Med. 2021;131(9):854-61. doi: 10.20452/pamw.16077

82. Green MF, Lee J, Wynn JK. Experimental approaches to social disconnection in the general community: can we learn from schizophrenia research? World Psychiatry. 2020;19(2):177-8. doi: 10.1002/wps.20734

83. Elbogen EB, Lanier M, Blakey SM, Wagner HR, Tsai J. Suicidal ideation and thoughts of self-harm during the COVID-19 pandemic: The role of COVID-19-related stress, social isolation, and financial strain. Depress Anxiety. 2021. doi: 10.1002/da.23162

84. Wasserman D, van der Gaag R, Wise J. The term "physical distancing" is recommended rather than "social distancing" during the COVID-19 pandemic for reducing feelings of rejection among people with mental health problems. Eur Psychiatry. 2020;63(1):e52. doi: 10.1192/j.eurpsy.2020.60

**Figures Legends**

Fig 1. Flowchart of the study

Fig 2. Prevalence of anxiety, depression, and post-epidemic stress symptoms in seven geographical regions of China.

Supplementary Fig 1. The sampling process of the units that recruited infertile men. The random selected hospitals conducted patient recruitment based on inclusion and exclusion criteria.
