# Supplementary material for: Psychological status of infertile men during the Coronavirus Disease 2019 Pandemic in China: a cross-sectional investigation
Source: Basic Clin Androl. 2023 Feb 16;33:8. doi: 10.1186/s12610-022-00177-5 (PMC9931448; doi:10.1186/s12610-022-00177-5)
Supplement: Supplementary file 2 — Additional file 2: Table 3. Descriptive Statistics of Sexual function and COVID-19 Related Information for the Total Sample. [file 12610_2022_177_MOESM2_ESM.docx]

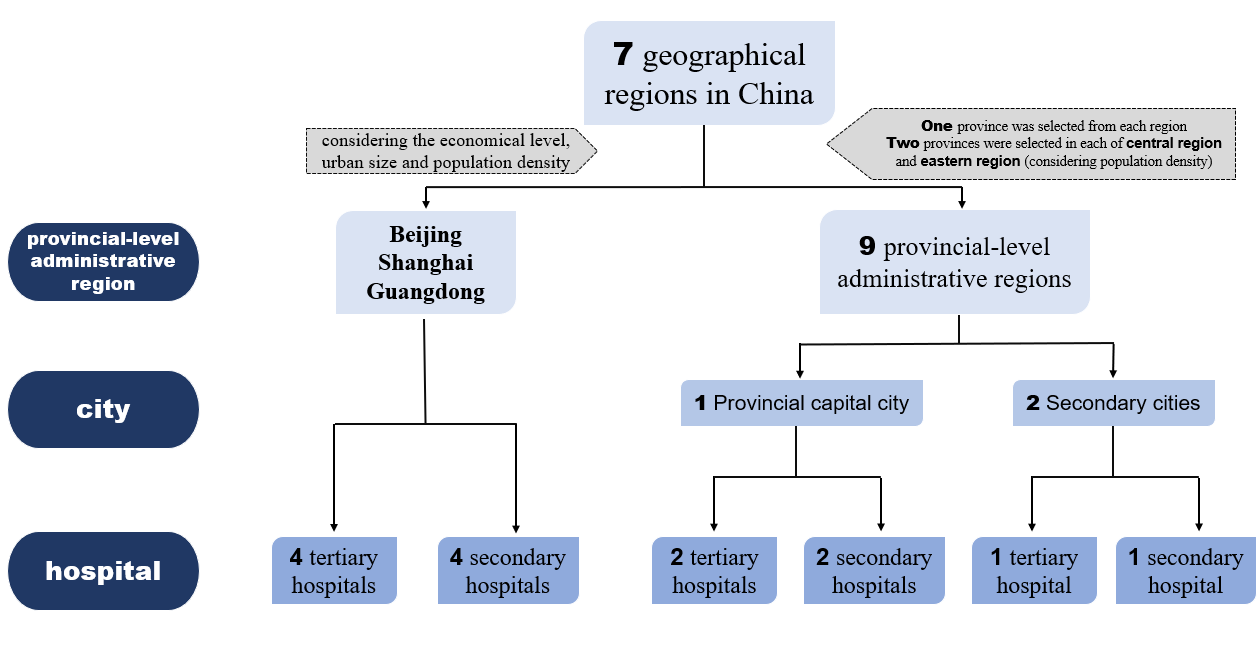


Supplementary Fig 1. The sampling process of the units that recruited infertile men. The random selected hospitals conducted patient recruitment based on inclusion and exclusion criteria.
