# Supplementary material for: Psychological status of infertile men during the Coronavirus Disease 2019 Pandemic in China: a cross-sectional investigation
Source: Basic Clin Androl. 2023 Feb 16;33:8. doi: 10.1186/s12610-022-00177-5 (PMC9931448; doi:10.1186/s12610-022-00177-5)
Supplement: Supplementary file 3 — Additional file 3: Table 2. Demographic information for the Total Sample. [file 12610_2022_177_MOESM3_ESM.docx]

**Supplementary Table 3. Descriptive Statistics of Sexual function and COVID-19 Related Information for the Total Sample**

| **Factors** | **Participants，No.** | **(%**^a^**)** |
| --- | --- | --- |
| **Overall** | 4098 | 100.0 |
| ***Sexual function*** | | |
| **Ongoing treatment** |  |  |
| None | 2405 | 58.7 |
| Medication | 1237 | 30.2 |
| Surgical treatment | 87 | 2.1 |
| Others | 369 | 9.0 |
| **ED symptom** |  |  |
| None | 1761 | 43.0 |
| Mild | 1343 | 32.8 |
| Moderate to severe | 994 | 24.3 |
| **PE symptom** |  |  |
| None | 3053 | 74.5 |
| Suspected | 432 | 10.5 |
| Yes | 613 | 15.0 |
| ***COVID-19 related information*** | | |
| **Did you get fever, fatigue or headache during pandemic?** | | |
| No | 4034 | 98.4 |
| Yes | 64 | 1.6 |
| **Are you a frontline worker?** |  |  |
| No | 3594 | 87.7 |
| Yes | 504 | 12.3 |
| **Have you ever experienced quarantine?** |  |  |
| No | 3305 | 80.6 |
| Centralized | 63 | 1.5 |
| Home | 730 | 17.8 |
| **How was your work affected by the pandemic?** |  |  |
| None | 1393 | 34.0 |
| Delayed | 842 | 20.5 |
| Kept jobless | 148 | 3.6 |
| Salary cut or job loss | 1438 | 35.1 |
| Workload increased | 277 | 6.8 |
| **Are you worried about you and your relatives infected by coronavirus?** | | |
| No | 2618 | 63.9 |
| Yes | 1480 | 36.1 |
| **Are you concerned that infection with coronavirus may affect your sexual function?** | | |
| No | 3247 | 79.2 |
| Yes | 851 | 20.8 |
| **Have you considered choosing cryopreservation of sperm in response to the COVID-19 pandemic?** | | |
| No | 3607 | 88.0 |
| Yes | 491 | 12.0 |
| **How much do you** **know about COVID-19?**  **(Rating from 0, not at all to 10, very well)** | | |
| 7.79±2.04 | - | - |
| **How much do you desire to receive psychological counseling?**  **(Rating from 0, not at all to 10, very well)** | | |
| 5.28±3.38 | - | - |

Abbreviation: ED, erectile dysfunction; PE, premature ejaculation; COVID-19, coronavirus disease 2019.

^a^: The proportion of the subgroup to the total.
