# Supplementary material for: Psychological status of infertile men during the Coronavirus Disease 2019 Pandemic in China: a cross-sectional investigation
Source: Basic Clin Androl. 2023 Feb 16;33:8. doi: 10.1186/s12610-022-00177-5 (PMC9931448; doi:10.1186/s12610-022-00177-5)
Supplement: Supplementary file 4 — Additional file 4: Table 1. Summary of First Three Parts of the Questionnaire. [file 12610_2022_177_MOESM4_ESM.docx]

**Supplementary Table 2. Demographic information for the Total Sample**

| **Factors** | **Participants，No.** | **(%**^a^**)** |
| --- | --- | --- |
| **Overall** | 4098 | 100.0 |
| **Age** |  |  |
| ＞32 | 1756 | 42.9 |
| ≤32 | 2342 | 57.1 |
| **BMI** |  |  |
| <18.5 | 168 | 4.1 |
| 18.5-24.9 | 2344 | 57.2 |
| >24.9 | 1586 | 38.7 |
| **Family composition** |  |  |
| Nuclear | 2476 | 60.4 |
| Extended | 1480 | 36.1 |
| Others | 142 | 3.5 |
| **Level of education** |  |  |
| Junior high school or below | 573 | 14.0 |
| Senior high school or technical secondary school | 896 | 21.9 |
| College degree or higher | 2629 | 64.2 |
| **Annual family income (¥, yuan)** |  |  |
| 0-49,999 | 994 | 24.3 |
| 50,000-99,999 | 1671 | 40.8 |
| ≥100,000 | 1433 | 35.0 |
| **Job status** |  |  |
| Erratic | 809 | 19.7 |
| Steady | 3289 | 80.3 |
| **History of psychiatric disorders** |  |  |
| No | 4016 | 98.0 |
| Yes | 82 | 2.0 |
| **Sleep disorders** |  |  |
| None | 3264 | 79.6 |
| Insomnia | 278 | 6.8 |
| Snoring | 480 | 11.7 |
| Both | 49 | 1.2 |
| Others | 27 | 0.7 |
| **Sleeping duration, per night** |  |  |
| <8h | 1654 | 40.4 |
| ≥8h | 2150 | 52.5 |
| Variable | 294 | 7.2 |

Abbreviation: BMI, Body Mass Index.

^a^: The proportion of the subgroup to the total.
